# Nek2 Kinase Signaling in Malaria, Bone, Immune and Kidney Disorders to Metastatic Cancers and Drug Resistance: Progress on Nek2 Inhibitor Development

**DOI:** 10.3390/molecules27020347

**Published:** 2022-01-06

**Authors:** Dibyendu Dana, Tuhin Das, Athena Choi, Ashif I. Bhuiyan, Tirtha K. Das, Tanaji T. Talele, Sanjai K. Pathak

**Affiliations:** 1 Chemistry and Biochemistry Department, Queens College of the City University of New York, 65-30 Kissena Blvd., Flushing, NY 11367, USA; dibyendu.iitm@gmail.com (D.D.); dtuhin26@gmail.com (T.D.); achoi8159@bths.edu (A.C.); ashif.qclab@gmail.com (A.I.B.); 2 KemPharm Inc., 2200 Kraft Drive, Blacksburg, VA 24060, USA; 3 Brooklyn Technical High School, 29 Fort Greene Pl, Brooklyn, NY 11217, USA; 4 Chemistry Doctoral Program, The Graduate Center of the City University of New York, 365 5th Ave, New York, NY 10016, USA; 5 Department of Cell, Developmental, and Regenerative Biology, Icahn School of Medicine at Mount Sinai, New York, NY 10029, USA; tirtha.kamal.das@gmail.com; 6 Mindich Child Health and Development Institute, Department of Pediatrics, Department of Genetics and Genomic Science, Icahn School of Medicine at Mount Sinai, New York, NY 10029, USA; 7 Department of Pharmaceutical Sciences, College of Pharmacy and Health Sciences, St. John’s University, 8000 Utopia Parkway, Queens, NY 11439, USA; talelet@stjohns.edu; 8 Biochemistry Doctoral Program, The Graduate Center of the City University of New York, 365 5th Ave, New York, NY 10016, USA

**Keywords:** Nek2 kinase signaling, Nek2 inhibitors, mitotic kinase, centrosomal kinase, Nek2 in cancer metastasis, Nek2 PROTACS, Nek2 kinase review, small molecule inhibitors of Nek2 kinase

## Abstract

Cell cycle kinases represent an important component of the cell machinery that controls signal transduction involved in cell proliferation, growth, and differentiation. Nek2 is a mitotic Ser/Thr kinase that localizes predominantly to centrosomes and kinetochores and orchestrates centrosome disjunction and faithful chromosomal segregation. Its activity is tightly regulated during the cell cycle with the help of other kinases and phosphatases and via proteasomal degradation. Increased levels of Nek2 kinase can promote centrosome amplification (CA), mitotic defects, chromosome instability (CIN), tumor growth, and cancer metastasis. While it remains a highly attractive target for the development of anti-cancer therapeutics, several new roles of the Nek2 enzyme have recently emerged: these include drug resistance, bone, ciliopathies, immune and kidney diseases, and parasitic diseases such as malaria. Therefore, Nek2 is at the interface of multiple cellular processes and can influence numerous cellular signaling networks. Herein, we provide a critical overview of Nek2 kinase biology and discuss the signaling roles it plays in both normal and diseased human physiology. While the majority of research efforts over the last two decades have focused on the roles of Nek2 kinase in tumor development and cancer metastasis, the signaling mechanisms involving the key players associated with several other notable human diseases are highlighted here. We summarize the efforts made so far to develop Nek2 inhibitory small molecules, illustrate their action modalities, and provide our opinion on the future of Nek2-targeted therapeutics. It is anticipated that the functional inhibition of Nek2 kinase will be a key strategy going forward in drug development, with applications across multiple human diseases.

## 1. Introduction

**Figure 1:**
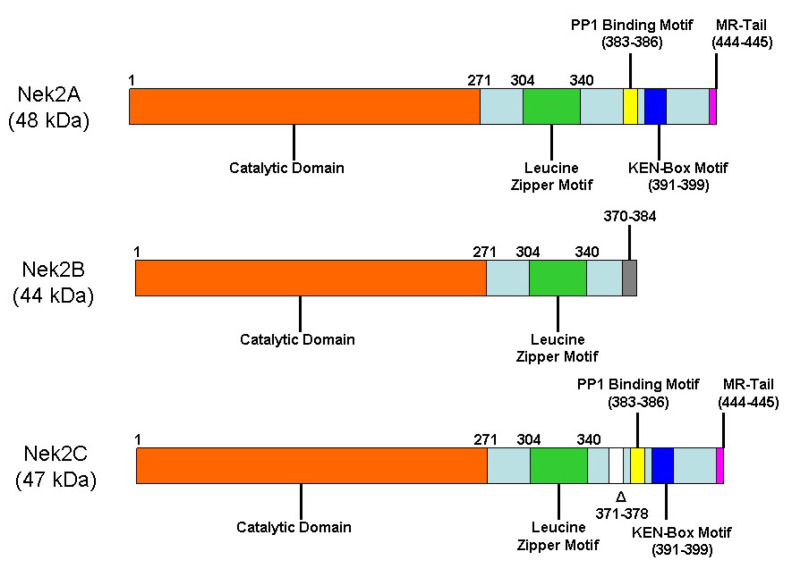
Structural organization of the three splice-isoforms of Nek2 kinase at the primary sequence level: Nek2A, Nek2B, and Nek2C. The three isoforms differ significantly at their C-terminus and are reported to play context-specific roles.

The human kinome represents the second most important class of therapeutic target after G-protein-coupled receptors (GPCRs). It is estimated that about 30% of all drug development projects in pharmaceutical companies are directed at kinases and their inhibition [1]. Not surprisingly, while as many as 76 protein kinase inhibitors are currently approved for clinical use by the United States Food and Drug Administration, several hundred are currently in the advanced stages of clinical development [2]. These levels of success in clinics make kinases one of the most promising druggable targets. The human genome encodes for eleven Never In Mitosis A (NIMA)-related serine/threonine protein kinases (Nek1-11) that share about 42% of the amino acid sequence identity with the amino-terminal catalytic domain of the NIMA kinase, a kinase originally identified in the fungus *Aspergillus nidulans* and shown to be essential for mitotic entry of the cell [3]. A temperature-sensitive mutant of the NIMA kinase was shown to block the cell’s entry into mitosis, resulting in cells being arrested in the G2 phase [4]. Human Nek2, a prominent member of the Nek family kinases and with most resemblance to the NIMA kinase, has emerged as a key player in many cellular signaling pathways. Three splice variant isoforms of the Nek2 kinase have been detected in human cells: Nek2A (48 kDa), Nek2B (44kDa), and Nek2C (47 kDa) (Figure 1) [5,6,7]. Interestingly, the non-centrosomal pools of all three Nek2 splice variants have unique cellular distributions; while Nek2A exhibits near uniform distribution in the cytoplasm and nucleus, Nek2B is primarily cytoplasmic, and Nek2C nuclear [8]. It should be noted that the context-specific functions of these variants in cell signaling have yet to be fully delineated. Nek2A, the full-length variant with 445 amino acid residues (hereafter referred to as Nek2), houses a N-terminus kinase domain and a C-terminus non-catalytic regulatory domain. In fully active form, Nek2 is a homodimer. This homo-dimerization is promoted by a leucine zipper (LZ) motif that yields a fully active homo-dimeric Nek2 kinase upon mutual auto-phosphorylation of several C-terminal residues [9]. Nek2 primarily localizes at centrosomes and has been shown to regulate centrosome separation and bipolar spindle formation. These two phenomena are central to mitotic chromosome segregation into two daughter cells; this is elegantly regulated by Nek2 kinase with extreme precision to achieve such a massive reorganization of cellular components. A loss of control over centrosomal machinery due to aberrant Nek2 function has been shown to produce chromosome segregation error with cells containing abnormal chromosome contents—a condition known as Aneuploidy, which is a primary cause of tumorigenesis [10,11,12,13]. In addition to its role as a centrosomal kinase, Nek2 also has other non-centrosomal biological functions [14,15,16]. In non-centrosomal roles, Nek2 participates in DNA damage response pathways in that it prevents the centrosome separation in cells containing damaged DNA [17]. Other non-centrosomal functions of Nek2 include spindle checkpoint control, chromatin condensation, the formation of the microtubule-organizing center (MTOC), mitotic redistribution of the Golgi apparatus, and cytokinesis [14,15,16]. Similarly, in mouse meiotic spermatocytes, Nek2 kinase activity was augmented by the components of the mitogen-activated protein kinase (MAPK) pathway [18]. 

High expression levels (2-5-fold above baseline) of the Nek2 kinase in a variety of highly aggressive solid tumors, including triple negative breast cancer, makes this kinase a high value target for developing anti-cancer drugs [19,20,21,22,23,24]. A few notable reviews have recently emerged that elegantly describe the current state of knowledge related to oncogenic Nek2 kinase signaling and inhibitor development [7,25,26]. The purpose of this review is to (a) highlight the critical role this kinase plays in the signaling of both normal cells and diseased cells involving polycystic kidney disorder, immune disorders, malarial infection, drug resistance, and activation of metastatic cancers, (b) discuss and analyze the small molecule inhibitors of the Nek2 kinase reported thus far, and (c) provide a rationale for the development of novel therapeutics targeting Nek2 kinase inhibition. Finally, our review also highlights the recent development of various whole animal models to study Nek2 function in order to more accurately capture the complex interplay between normal and diseased cells in the progression of the disease and to identify drugs with an optimal therapeutic index.

## 2. Nek2 Signaling in Normal Human Physiology

Mitosis is a critically important cellular process that must be regulated with exquisite precision. Nek2 is involved in the disassembly of the tether (inter-centriolar linker) at the onset of mitosis to facilitate the centrosome separation that follows the establishment of the mitotic spindle (reviewed by Fry et al. [27]). Although how precisely Nek2 regulates these events is still not completely clear, current literature suggests that phosphorylation of linker proteins, cNAP1, and rootletin by the Nek2 kinase helps in the tether dissolution [28,29,30]. Other components of this linker such as β-catenin and Cep68 proteins are also regulated by Nek2 kinase activity [23,31]. 

**Figure 2:**
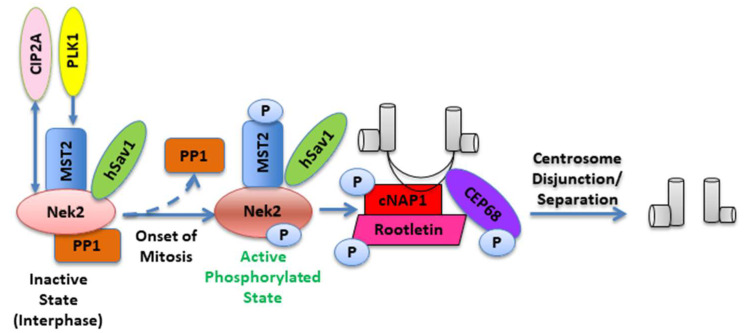
Role of upstream signal transduction regulators of Nek2 kinase in centrosome disjunction and separation. During interphase stage of the cell cycle, Nek2 remains in an inactive state as a complex of PP1 and hippo pathway regulators MST2/hSav1 via SARAH domain interactions (Mardin, Fry, Nature Cell Biology 2010); this is mediated through PP1 dephosphorylation antagonism. At the onset of mitosis, activated PLK1 phosphorylates MST2 kinase, thereby releasing the PP1 antagonism switch and promoting active phosphorylated Nek2 kinase enrichment at the centrosome. Active Nek2 then phosphorylates centrosome linker proteins, cNAP1 and rootletin, and mediates centrosome disjunction and separation. Another important regulator of Nek2 kinase is CIP2A, which directly binds and enhances Nek2 activity, independent of PP1 involvement.

During interphase of the cell cycle, Nek2 exists as a complex with protein phosphatase 1 (PP1) and mammalian STE20-like protein kinase 2 (MST2), an important component of the Hippo pathway; it is maintained in an inactive state through dephosphorylation by PP1 [29,32]. Polo-like Kinase 1 (Plk1), a serine-threonine kinase, is an important regulator of various stages of the cell cycle including centrosome maturation, spindle assembly, kinetochore formation, mitotic exit, and cytokinesis [33,34]. At the onset of mitosis, Plk1-mediated phosphorylation of MST2 prevents PP1 from binding to the MST2-Nek2 complex [35]. This enables Nek2 to undergo autophosphorylation and thereby become active. The activated Nek2 subsequently phosphorylates the inter-centriolar linker proteins cNAP1 and rootletin, causing their displacement from the centrosome (Figure 2) [36,37]. In a recent study, Jeong et al. identified Nek2 as a binding partner for cancerous inhibitor of protein phosphatase 2A (CIP2A) [38]—a protein involved with uncontrolled cancer cell proliferation in a large variety of human malignancies [39]. Analysis of their data showed that the N-terminal kinase domain of Nek2 binds and interacts with the C-terminal coiled-coil domain of CIP2A at the G2/M transition. More importantly, CIP2A was found to be a key modulator of Nek2 activity, independent of PP1 (or PP2A) pathway regulation; these findings posit Nek2 as an essential target for CIP2A-induced tumorigenesis [38]. In another important study, Pitner et al. sought to identify the causal factors of centrosome amplification (CA), an established promoter of carcinogenesis, aneuploidy, and chromosomal instability, in Her2+ breast cancer models [40]. They demonstrated a molecular connection between Cdk4 and Nek2, as downregulation of one protein negatively impacted the expression levels of the other; a direct correlation between Nek2 expression levels and centrosome amplification and binucleation was also observed. This important study suggests that Nek2 is a potential inhibitory target for mitigating CA in Her2+ breast tumors. 

**Figure 3:**
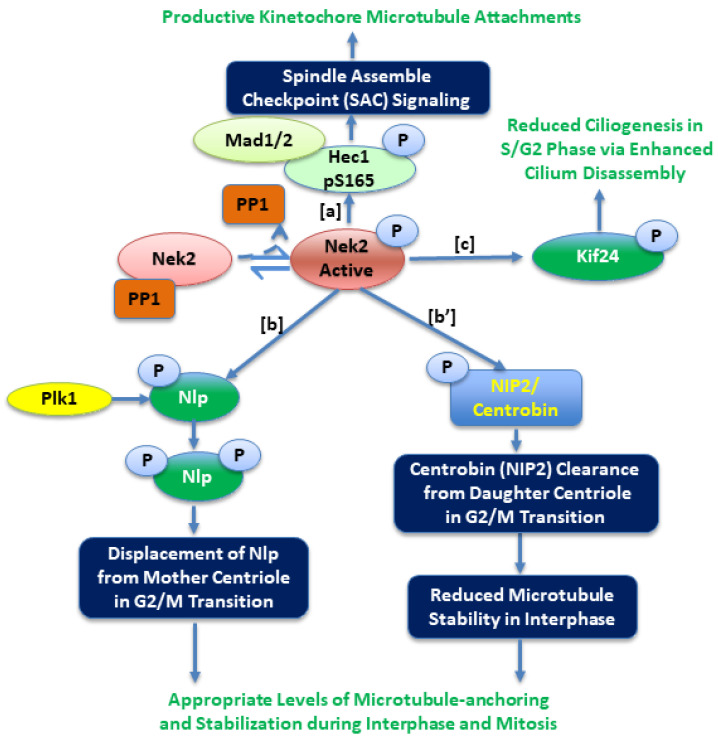
The role of Nek2 kinase in spindle assembly checkpoint (SAC) signaling, microtubule dynamics during G2/M transition, and ciliogenesis: (a) Nek2 phosphorylates Hec1 at Ser165, preferentially at the kinetochores during SAC. Hec1-pS165 then recruits and interacts with MAD1 protein to promote SAC signaling on misaligned chromosomes lacking proper microtubule attachment. (b, b’) During G2/M transition, Nek2 kinase also regulates (via phosphorylation) the displacement of (i) mother centriole component protein, Nlp, and (ii) daughter centriole component protein centrobin (NIP2). This leads to appropriate levels of microtubule anchoring and stabilization during interphase and mitosis. (c) Nek2 kinase activates microtubule depolymerizing kinesin protein, Kif24, via direct phosphorylation and negatively regulates ciliogenesis in proliferating cells.

Experimental evidence suggests that Nek2 localizes to the kinetochore and plays an important role in spindle assembly checkpoint (SAC) signaling for faithful chromosome segregation by mediating interactions with Hec1 and MAD1 proteins (Figure 3) [41,42,43]. During this critical process, the appropriate timing of proper microtubule attachments to kinetochores is one of the key determinants (else mitotic arrest). Studies show that this event, regulated by the levels of Hec1-S165 phosphorylation, is carefully orchestrated by the Nek2 kinase [43,44]. Indeed, the phospho-mimicking Hec1 mutant (Hec1S165E) perturbed the chromosome alignment and triggered severe mitotic arrest associated with Mad1/Mad2 downstream signaling [43]. Not surprisingly, disruption of the Hec1/Nek2/Mad1/Mad2 axis by small molecules imparted impressive anticancer activities [45,46,47]. Microtubule reorganization at the G2/M transition requires major changes in cytoskeletal dynamics, many resulting from post-translational modifications. To coordinate this complex event, several centrosomal proteins must be recruited and/or displaced or degraded at appropriate times [48,49,50]. During interphase, a γ-tubulin-binding ninein-like protein (Nlp)—a mother centriole-specific protein—is present at the centrosome but is displaced from it upon mitotic entry [51]. The Nlp protein has been shown to be a substrate of both Nek2 and Plk1 kinases [24]. Rapley et al. showed that overabundance of either the Nek2 or Plk1 kinase can result into its premature displacement from the centrosome during the interphase. They also demonstrated that initial phosphorylation of Nlp by the Nek2 kinase promoted its further phosphorylation by the Plk1 kinase, suggesting that Nek2 is an upstream regulator of Nlp function [24]. Another important player in this G2/M transition is a daughter-centriole-specific protein, centrobin (Nek2-interacting protein 2 (NIP2)), which is also a subject of post-translational regulation by both the Nek2 kinase and Plk1 kinase at distinct phosphorylation sites [52,53]. It is known that for proper assembly of functional mitotic spindle, centrobin (NIP2) function is essential [54]. In an important study, Park at al. concluded that centrobin phosphorylation by the Nek2 kinase antagonized its microtubule stabilizing function during interphase [53]. Consistently, while Nek2-depleted cells appeared to have enhanced migratory behavior and a well-developed microtubule network with higher stability, centrobin-depleted cells exhibited the opposite phenotypes. Once the cell enters mitosis, however, Plk1 phosphorylates centrobin and synergizes its function, resulting in higher microtubule stability with accurate bipolar spindle formation.

Primary cilium, a microtubule-based projection from the cell surface, plays critical communication roles in quiescent and differentiated cells in response to external stimuli [55]. Cell cycle and cilium biogenesis are reciprocally regulated, and the length of cilia serves as a physical block for cell cycle progression [56]. Upon receiving mitotic signals, primary cilia must be disassembled and resorbed. Histone deacetylase 6 (HDAC6), HEF1, the Aurora A kinase, and the Nek2 kinase play an important role in the cilium disassembly process [57,58]. In an elegant functional study, Kim et al. reported that a microtubule depolymerizing kinesin protein, Kif24, colocalizes with the Nek2 kinase at the S/G2 phase and is phosphorylated (Figure 3). Phospho-Kif24 exhibits enhanced microtubule depolymerizing activity, inhibiting the cilia growth in proliferating cells [59]. Viol et al. showed that Nek2 activity is required for the release of a subset of distal appendages (DAs), including Cep123, Cep164, and LRRC45 from the mother centriole at the onset of mitosis [60]. In the absence of the Nek2 kinase, ciliary disassembly is incomplete and leads to the persistence of a ciliary remnant at the older centrosome of mitotic cells; this facilitates cilia elongation shortly after division. Recent work by DeVaul et al. has established that cilia growth or shortening depends on the novel functional interactions between the Nek2 and AurA kinases [61]. When cilia were growing, either Nek2 kinase or AurA kinase activities were able to independently shorten it. However, when they were being absorbed, both Nek2 and AurA activities were indispensable. Additionally, there were synergistic interactions between the two kinases during cilia assembly but not disassembly. While inhibition of AurA enhanced cilia number, inhibition of Nek2 significantly stimulated cilia length. Finally, they also showed that a PP1 binding protein, PPP1R42, directly inhibited Nek2 function (independent pf PP1), and thus promoted ciliation in ARPE-19 cells. These studies together suggest that Nek2 is as an important kinase that controls DAs and the timing of cilia formation. 

## 3. The Nek2 Kinase in Polycystic Kidney Diseases (PKDs), Chromosomal Instability and Drug Resistance, Bone Destruction, and the DNA Damage Response Pathway

Polycystic kidney diseases (PKDs) are genetic disorders that primarily affect kidney function in both adults and children; they may also affect related organs such as the liver and pancreas. Kidneys in PKD contain multiple fluid-filled cysts that results from uncontrolled cell proliferation due to aberrant cilium signaling (for a review, see Bergmann et al [62]). Using a mouse kidney epithelial cell line model, Mahjoub et al. showed that Neks (mNek1 and mNek8; mutations in mNek1 and mNek8 leads to cystic kidneys) play critical roles in coordinating regulation of cilia signaling and cell cycle [63,64]. They established that while mNek1 localized to centrosomes in the interphase and remains associated with the mitotic spindle pole during mitosis, mNek8 was found to be near the proximal region of the primary cilium but not in the mitotic cells. Given that the Nek2 kinase has been intricately involved in cilia biogenesis, perhaps a direct link may exist between aberrant Nek2 function and PKDs [59,61,65,66,67]. 

Seminal work by Zhou et al. showed that Nek2 expression was highly correlated with drug resistance, resulting in poor prognosis of multiple cancer types [68]. This study also showed that Nek2 overexpression led to activation of Akt (with concomitant inhibition of PP1 via the direct phosphorylation of PP1 at Thr-320 by the Nek2 kinase [32]) signaling pathway, a finding also discovered independently by our group [69]. Akt activation steps involve phosphorylation first at Thr-450, followed by the phosphorylation at Thr-308 and Ser-473 residues; PP1 can dephosphorylate Thr-450 and deactivate Akt [70]. In addition, they also observed canonical Wnt signaling pathway activation, as evident from the accumulation of nuclear β-catenin and enhanced chromosomal instability (CIN). Two main mechanisms of drug resistance are the upregulation of mitotic checkpoint proteins and efflux pumps [71]. Overexpression of Nek2 stimulated (and downregulation inhibited) the expression of mitotic check point protein MAD2 and the ABC transporter family of proteins, ABCB1 (p-glycoprotein, MDR1), ABCC1 (MRP1), and ABCG2. Collectively, this study concluded that overabundant Nek2 kinase enhances pAkt signaling, resulting in the upregulation of efflux pump expression via downstream targets PIM1 and NF-kB; this leads to cancer drug resistance. These findings about the activation of Akt and drug resistance were consistent with findings from other laboratories [72,73]. Yang et al. showed that while aldehyde dehydrogenase 1-A1 (ALDH1A1) overexpression in myeloma cells led to overexpression of the Nek2 kinase both at the mRNA and protein levels with enhanced drug resistance, knock down of Nek2 expression by shRNA decreased drug efflux pump activity reducing drug resistance [74]. Similarly, a study by Marina et al. suggested that Nek2 and Plk4 might act in a synchronous manner to promote breast tumorigenesis and tamoxifen and trastuzumab resistance [75].

Noting that higher expression of the Nek2 kinase was directly correlated with bone lytic phenotypes in multiple myeloma patients, Hao et al. investigated its role in bone remodeling [76]. Overexpression of the Nek2 kinase in human bone marrow macrophages augmented osteoclast differentiation and bone loss. Interestingly, a positive correlation was observed in levels of Nek2 and an endoglycosidase enzyme, heparanase (HPSE), capable of cleaving heparan sulfate. Small molecule heparanase inhibitor, Roneparstat, dramatically reduced bone loss induced by Nek2 overexpression in an MM mouse model. This study posits a role for the Nek2 kinase in signaling involving bone remodeling. The way in which, at the molecular level, Nek2 overexpression leads to the upregulation of heparanase in bone metabolism still needs to be investigated. 

**Figure 4:**
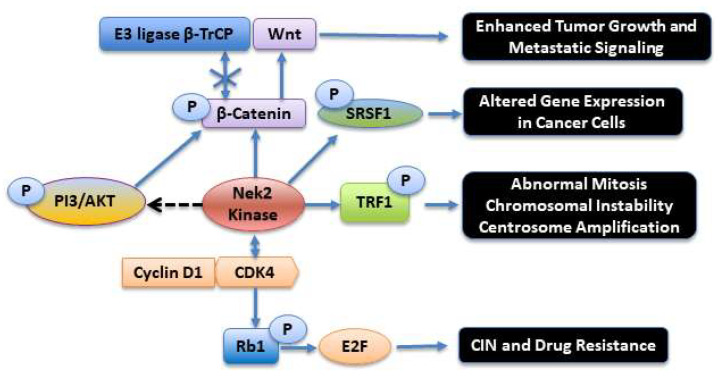
Aberrant Nek2 function promotes several key oncogenic signaling pathways.

Faithful DNA replication during cell division and its maintenance throughout the life span of an organism are critically important events. Chemical damage to DNA can, however, occur during the normal life span via several mechanisms, such as a reaction with radiation-induced free radicals, exposure to environmental toxins, and reactive oxygen species (ROS) [77,78]. Damaged or mutated DNA can initiate neoplastic transformation. Fortunately, several conserved DNA damage repair (DDR) pathways exist that can rectify undesirable chemical modifications to DNA and help maintain its integrity. When DNA damage is detected, specific checkpoint protein complexes play a well-orchestrated role in arresting the cell cycle at various stages and subsequently initiating the DNA repair processes [79,80,81,82,83]. One of the most deleterious DNA damage lesions is the double-strand break (DSB), which initiates a host of protein phosphorylation events, carefully orchestrated by protein kinases, for the repair [84]. When a DSB is detected, the ataxia telangiectasia mutated (ATM) kinase, a Ser/Thr kinase from the PI-3 kinase family, phosphorylates and activates several downstream cell cycle-regulated protein kinases in the DDR pathways [85]. Activation of several members of the Nek family has also been reported at various levels in DDR pathways, elegantly reviewed recently by Pavan et al. [86]. In the context of Nek2, it was observed that IR radiation of HeLa cells led to reduced Nek2 activation and centrosome splitting, leading to cell cycle arrest in G2 phase [17,87]. Interestingly, the reduced Nek2 activation was correlated well with ATM activation upstream. Activated ATM led to the upregulation of PP1 phosphatase activity (by the removal of inhibitory Thr320 phosphorylation) [88]. Since PP1 is also a negative regulator of Nek2 activity, this led to the inhibitory modulation of Nek2 function, such as in centrosome splitting. Nucleophosmin (NPM), a multifunctional protein regulating cell proliferation and cell death, is phosphorylated at Ser70 and Ser88 by the Nek2 kinase [89,90]. Under normal physiological conditions, phosphoNPM interacts with a highly important tumor suppressor protein, alternate reading frame (ARF). PhosphoNPM-ARF interaction is critical for the tumor suppressor function of ARF. Upon IR-induced DNA damage, which results in the activation of ATM and PP1, the Nek2-induced phosphorylation of NPM is downregulated. This results in the dissociation of ARF from NPM, and its subsequent degradation by ULF E3-ubiquitin ligase [90,91]. Another important protein that Nek2 binds to and phosphorylates is telomeric repeat binding factor 1 (TRF1) [92,93]. TRF1 is a double-stranded telomere DNA-binding protein that plays an important role in cell cycle regulation and in promoting efficient DNA replication at telomere [94]. In one study, Nek2 over-expressions in MDA-MB-231 and MCF7 breast cancer cells resulted in mitotic aberrations such as abnormal centrosome contents and multinucleated cells. These phenotypes were contingent upon the presence of TRF1. Silencing of TRF1 prevented mitotic failure; a rescue experiment in cells, consisting of the addition of exogenous TRF1 to Nek2-overexpressed cells with endogenous TRF1 depletion, re-induced cytokinetic failure. Taken together, these results suggest that TRF1 is indispensable for overexpressed Nek2 to trigger abnormal mitosis and chromosomal instability (Figure 4) [92].

## 4. The Nek2 Kinase in Oncogenesis and Metastatic Signaling

Cell cycle kinases are considered attractive targets for the development of anti-cancer agents [95,96,97,98,99,100,101,102,103]. This includes members of the Nek family of mitotic kinases, known to play key roles in centrosomal duplication, spindle formation, and chromosomal segregation (elegantly reviewed by Fry et al. [27,104,105]). The critical role of the Nek2 kinase in coordinating the centrosomal cycle and the microtubule organization and stabilization in preparation for mitosis is now well established. Aberrant regulation of Nek2 kinase activity has been associated with several phenotypical hallmarks of cancer, such as centrosome amplification, chromosomal instability, and abnormal cell proliferation [7,69,106,107,108]. Indeed, Nek2 expression pattern is abnormally high in many different types of cancers, both at the mRNA and protein levels [21,68,109,110,111,112,113,114,115,116,117,118]. When activated either by extra- or intra-cellular stimuli (e.g., cell–cell contact, mechanical stress signals, and cell polarity or stiffness of the extracellular matrix), Hippo signaling is activated and exerts a critical control in modulating cell growth and proliferation, thereby affecting the progression of diseases like cancer and drug resistance [119,120,121,122,123]. The principal components of the mammalian Hippo pathway network involves kinase signaling cascades, orchestrated by two Ser/Thr kinases, Mammalian Ste20-like 1 and 2 (MST1/2) and Large Tumor Suppressor 1 and 2 (LATS1/2). Two adaptor proteins containing the WW-domain interactions are also involved—the Salvador (SAV1) protein interaction with the MST1/2 kinase and the Mps One Binder 1 (MOB1) protein interaction with the LAT1/2 kinase. A “turned on” Hippo pathway results in the phosphorylation and thus the activation of the LATS1/2-MOB1 protein complex by the MST1/2-SAV1 complex. The downstream effect of this event is phosphorylation of the YAP/TAZ protein complex that enhances its cytosolic retention (and thus reduced nuclear localization) and proteasomal degradation. A diminished presence of dephosphorylated YAP/TAZ in the nucleus does not allow YAP/TAZ to form a productive transcriptional complex with nuclear protein TEA domain (TEAD) proteins, thus inhibiting the expression of the Hippo-responsive genes responsible for cell proliferation, migration, and survival [124]. Recent findings in fruit flies suggest that during normal development, Anaphase-Promoting Complex/Cyclosome (APC/C), a key E3 ubiquitin ligase with an established role in cell cycle progression, in a complex with Cdh1/Fzr, downregulates Nek2 kinase activity via degradation [125]. Dishevelled (Dsh), a protein responsible for promoting epithelial planar cell polarity (PCP), is a direct substrate of the Nek2 kinase and when phosphorylated also undergoes degradation by the APC/C/Cdh1 complex [126]. So when Nek2 is overabundant or upregulated, it could lead to downregulation of Dsh, thereby directly affecting PCP signaling pathways, such as in malignant tumors. In accordance with this, Kim et al. subsequently showed that the APC/C/Cdh1 complex can also degrade LATS kinases (and hence enhance the nuclear localization of YAP/TAZ), thereby turning on genes involved in the cell proliferation pathway [127]. 

Another evolutionary pathway often dysregulated in tumors where the Nek2 kinase is involved is Wnt signaling pathway. A key player in this pathway is β-catenin, a multifunctional protein involved in key cellular functions, such as centrosome disjunction, bipolar spindle formation, and cell-cell adhesion [128,129]. It is now well established that Wnt/β-Catenin signaling is aberrant in many kinds of highly invasive cancers [130,131,132,133]. In a highly important study, Bahmanyar et al. showed that β-catenin is a physiological substrate of the Nek2 kinase and is a key player in the intercentrosomal linker complex, regulating centrosome separation during mitosis [23]. In a subsequent study, Mbom et al. investigated how β-catenin is regulated at the centrosome [134]. They found that the Nek2 kinase phosphorylates β-catenin at a N-terminal phosphorylation site that is in the same regulatory domain as in glycogen synthase kinase 3β (GSK3β). This phospho-β-catenin is stabilized by lack of affinity toward the E3 ligase β-TrCP that prevents β-catenin ubiquitination and degradation (Figure 4). They also show that polo-like kinase 1 (Plk1) is an upstream kinase that regulates Nek2 phosphorylation and hence activation. Several studies have emerged in which downregulation or inhibition of the Nek2 kinase function has resulted in the attenuation of Wnt/β-Catenin signaling in several types of cancers [135,136,136,137].

**Figure 5:**
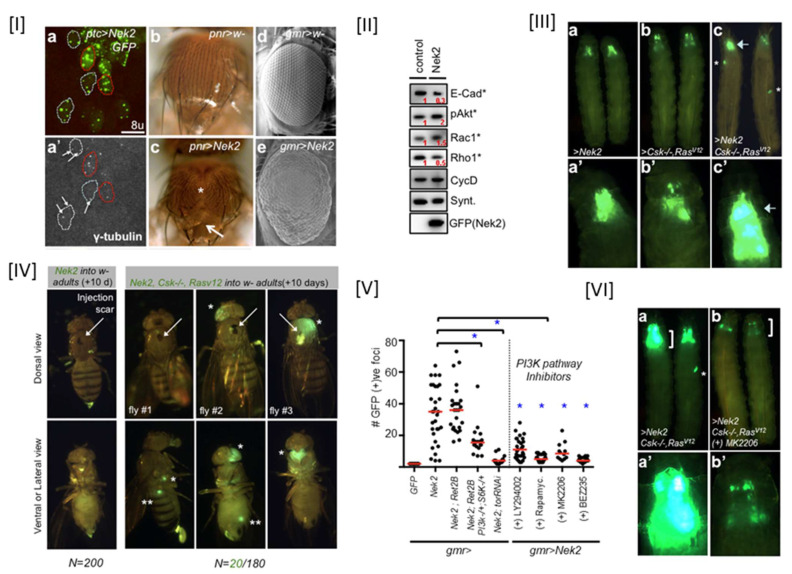
Development of Nek2 overexpression model in *Drosophila melanogaster*, its phenotypical evaluation, and utilization in studying signaling pathways: [**I**] (a and a’) Overabundance of dNek2 leads to centrosome amplification, as visualized by centrosomal marker γ tubulin (red circle, single white arrow). (c) Nek2 overexpression in thorax, driven by *pnr* promoter, results in defective notum and scutellum (asterisk), compared to wt flies, as in b. (e) Nek2 overexpression in eyes driven by *gmr* promoter results in patterning defects, compared to wt flies, as in d. [**II**] Western blot analysis suggests that, upon Nek2 overexpression, (i) key markers of cell migration pathway are dysregulated and (ii) PI3K/Akt pathway is activated. [**III**] (c) Co-expression of Nek2 kinase with oncogenic Ras (Csk−/−, Ras^V^ [12]) promotes primary tumor mass increase and distant cell seeding phenotype (asterisk), compared to Nek2 kinase alone as in a and oncogenic Ras alone as in b (eye-FLP-MARCM experiment). [IV] Tumor cell injection assay indicates the close cooperation of Nek2 kinase signaling with oncogenic Ras to promote migration of primary tumor foci away from the tumor injection site (asterisks), compared to control (Nek2 in w-adults). [**V**,**VI**] Genetic and pharmacological inhibition of PI3K pathway significantly reduces cell seeding phenotypes in Nek2 overexpression flies, as well as primary tumor growth in b, compared to control a. [Adopted from Das et al., Oncogenesis 2013, 2, e69, with permission].

While several highly invasive cancers have been documented to overexpress the Nek2 kinase, the molecular mechanism by which its overabundance promoted tumorigenesis, as well as the question as to whether this played any role in invasion and metastatic signaling, was unknown. Since cancer models in cell-based systems do not mimic a real tumor microenvironment, a whole animal-based Nek2 overexpression model was needed to understand the complexities of the signaling events [138]. We therefore developed a *Drosophila melanogaster* model where dNek2 overexpression with a GFP tag was achieved in a tissue-specific manner: eye, thorax, and wing (Figure 5) [69]. dNek2 overexpression led to centrosome amplification, defective notum and scutellum, and abnormal patterning in eyes. Another important and highly interesting phenotype observed in these flies was distant seeding—a process that indicated cell migration from overabundant dNek2 tissues to the other parts of the body. Molecular level investigation using Western Blot analysis further revealed that dNek2 overexpression activated PI3K/Akt signaling and promoted the Wnt ortholog wingless (Wg) pathway with alterations of several cell migration markers such as Rho1, Rac1, and E-cadherin. These results together strongly suggest that overexpression of the dNek2 kinase enhances cell survival and migration and metastatic phenotypes. Since cancer is often a multigenic disease, we tested a hypothesis that the dNek2 kinase could cooperate with other oncogenes to promote invasion and metastasis. If true, Nek2 can then become an attractive anticancer target using network polypharmacological approaches [139]. Overexpression of the dNek2 kinase with either oncogenic Ras (*Csk−/−; Ras^V^*^12^) or oncogenic RET (*dRet^MEN^*^2^) resulted in enhanced survival and aggressive metastasis, and this cooperativity relied on synergistic PI3K/Akt signaling. Using a quantitative assay on a distant seeding phenotype, we further showed that both genetic and pharmacological inhibition of the components (e.g., PI3K, mTor, Akt) of the PI3K/Akt pathway reduced metastasis. Finally, using this Nek2 fly model, we discovered a novel non-toxic quinoline-based pharmacophore that inhibited Nek2 function both *in vitro* and *in vivo* (see Section 6, Table 1, Entry 8).

## 5. Global Proteomic and Phosphoproteomic Data and Activation of the Nek2 Kinase in Cancers

Tumor heterogeneity is one of the major obstacles in implementing proper treatment protocol in cancers. To overcome this challenge, researchers are now taking advantage of the Clinical Proteomic Tumor Analysis Consortium (CPTAC) data sets and gaining an overall understanding of proteogenomics at a systems level. This database contains mass spectrometry-based proteomic and phosphoproteomic data sets conducted on human tumor samples from different origins. Taking advantage of this, Deb et al. recently mined and analyzed proteomic and phosphoproteomic data sets of tumor samples derived from six different cancers types: ovarian cancer, breast cancer, clear cell renal cell carcinoma (CCRCC), uterine corpus endometrial carcinoma (UCEC), colon cancer, and lung adenocarcinoma (LUAD) [158]. Using a combined integrative and bioinformatics approach, they identified 880-phosphopeptide signatures for differentially regulated phosphorylation sites across all but CCRCC cancer types. Their analysis revealed that the cell cycle was aberrantly activated across all these five types of cancers. Their effort then focused on identifying cell cycle kinases that are commonly activated across these five cancers; this would result in developing anti-cancer agents with broad efficacy for these cancers. Interestingly, they discovered that the Nek2 and Aurora Kinase A are the most activated kinases across all five cancer types. This finding suggests that drugs developed for either one of the two kinases could be repurposed for treatment across any of the five cancer types. 

## 6. Nek2 Ortholog in Malaria

**Figure 6:**
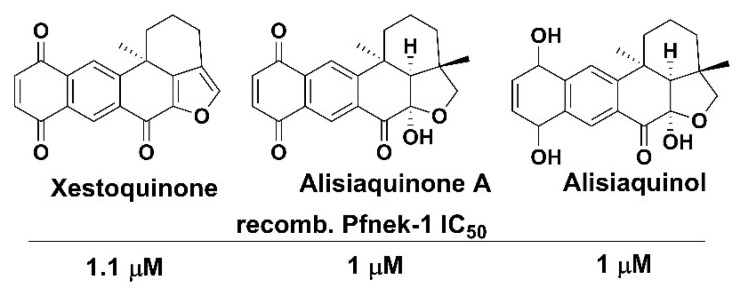
Quinone-based inhibitors of Pfnek-1 enzyme, derived from the deep-water marine sponge *Xestospongia* sp.

In 2019 alone, the World Health Organization (WHO) estimated 229 million cases of malaria worldwide with an estimated number of deaths of 409,000 [159]. Children under five were, notably, the most vulnerable group, accounting for an enormous 67% of all malarial deaths. The protozoan parasite *Plasmodium falciparum* is primarily responsible for morbidity and mortality in human malaria and is considered a valuable target for the development of effective therapeutics. Unfortunately, several resistance mechanisms to the existing drugs have emerged, making it a real threat to human life, especially in developing countries [160,161,162,163,164]. The anti-malarial vaccine development has also not been successful so far. Novel targets are therefore urgently needed, so effective strategies can be adopted to control this deadly disease. Interestingly, protein phosphorylation by several dozen kinases is known to play key roles in the progression and survival of the *Plasmodium* via both mosquito vector and human host, making them interesting new targets for drug development [165,166,167,168]. Among important kinases, there are four NIMA-related kinases, Pfnek-1, -2, -3, and -4, with Pfnek-1 being the closest homologue of the human Nek2 kinase [169,170]. While Pfnek-2, -3, and -4 are exclusively expressed in gametocytes, Pfnek-1 is expressed in both asexual and gametocyte stages of *Plasmodium’s* life cycle [171,172,173]. Using a reverse genetic approach, Dorin-Semblat et al. showed that the presence of Pfnek-1 is absolutely essential for the asexual cycle of the parasite in red blood cells [171]. They also showed that it is expressed only in male gametocytes, in contrast with Pfnek-2 and Pfnek4, thereby suggesting a non-redundant function of Pfnek-1. Importantly, as a proof-of-concept, they utilized the recombinant Pfnek-1 enzyme and screened a medium-sized small molecule library and identified several lead inhibitory compounds (data not shown) that could potentially be utilized for antimalarial drug development. Laurent et al. isolated xestoquinone (Figure 6) from a Vanuatu marine sponge *Xestospongia* sp. that showed good antiplasmodial activity against an FCB1 *P. falciparum* strain (IC_50_: 3 μM); it was also active *in vivo* at 5 mg/kg in *P. berghei* NK65 infected mice [174]. A similar study involving the New Caledonian deep-water sponge, Desoubzdanne et al. discovered two compounds, alisiaquinones A and alisiaquinol (Figure 6), that inhibited Pfnek-1 activity in the micromolar range (IC_50_: 1 μM) [175]. Further studies are needed to discover compounds with higher Pfnek-1 potency (IC ~ single digit nM range) and selectivity and to assess their anti-malarial potential in both normal and resistant strains of *P. falciparum*. 

## 7. Small Molecule Inhibitors of the Human Nek2 Kinase

For almost three decades now, protein kinases have garnered much-coveted attention as potential drug targets for various human diseases. This effort has culminated in more than 70 FDA-approved small molecule drugs so far. The era of kinase drug discovery was started by the discovery of staurosporine—a pan-kinase inhibitor—which, despite its large structure, outcompetes ATP and occupies the ATP-binding site [176,177]. Despite its promiscuous inhibitory profile, staurosporine provided a wealth of information on kinase-ligand interactions and established the ATP-binding site as a plausible target for developing kinase inhibitors. Further advancement in this area enabled the identification of other proximal binding sites along with the different modes of inhibitory mechanism that led to the discovery of a wide array of chemotypes with superior target selectivity. These chemotypes, depending on their binding interactions within the kinase pockets and their mode of inactivation, have been categorized in distinct classes, as described below:

(a) Type I: Chemotypes that occupy the ATP-binding site of an active kinase; this does not mandate specific conformation of key structural elements, such as αC helix or the DFG/Mg^++^ binding site, and allows interaction with the hinge region via H-bonding [178,179].
(b) Type I 1/2: Chemotypes that inhibit the ATP-binding site of an inactive target kinase with the DFG motif’s Asp ‘in’ and αC helix ‘out’ conformation [179,180].
(c) Type II: Chemotypes that occupy the ATP-binding pocket of an inactive kinase with DFG ‘out’ conformation [178,179].
(d) Type III: Allosteric modulators that bind within the cleft, formed by the small and large lobes, adjacent to ATP-binding pocket [178,179,181].
(e) Type IV: Allosteric modulators that bind next to ATP-binding pocket; however, unlike type III inhibitors, they bind outside of the cleft and the phosphoacceptor region [179,181].
(f) Type V: Chemotypes that occupy two distinct sites at once. This type of inhibitor has been further divided into two subcategories. These are (i) Bisubstrate analog inhibitors, which span over the ATP and substrate-binding site and (ii) Bivalent inhibitors, which span over the ATP-binding site along with any other site on the protein except at the substrate binding site [179,182].
(g) Type VI: Chemotypes with a built-in electrophilic warhead that trap the accessible nucleophilic protein residue to form a covalent adduct with the target kinase [179].

Nek2 has invigorated attention in recent years due to its involvement in several pathophysiological conditions. The biological implications of Nek2 inhibition have primarily been investigated using various biochemical techniques, such as the RNAi knockdown model. Although this approach excavated important findings on Nek2 kinase function, it did not mimic therapeutic inhibition of the target by the small molecules. This has inspired the discovery of several chemotypes in recent years for sensitizing Nek2 activity both *in vitro* and *in vivo*. Furthermore, another class of chemotype, a protein-protein interaction (PPI) inhibitor, which attenuates Nek2 protein activity by disrupting its interaction with Hec1 protein, has also emerged. In the following section, we discuss the emergence and advancement of the different classes of Nek2 inhibitors, categorize them to specific inhibitor types, and outline the possible impact of Nek2-selective inhibitory chemotype development on future drug discovery efforts.

## 8. ATP-Site Binding Inhibitors of Nek2

### 8.1. Pyrrole-Indoline-Based Ligand

**Figure 7:**
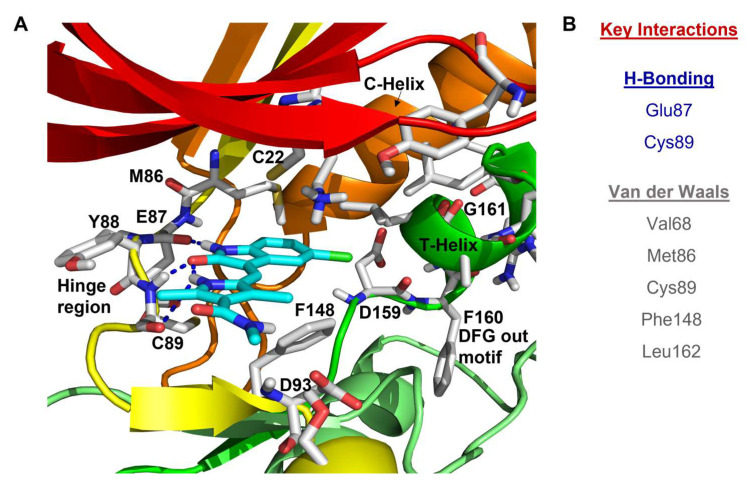
(**A**) SU11652 inhibits Nek2 by occupying the backside of ATP-binding cleft and forming a network of polar and non-polar interactions with the vicinal amino acid residues of the protein. (PDB code: 2JAV). (**B**) The enlisted amino acid residues engage with the ligand using both polar (H-bonding) and non-polar (van der Waals) interactions.

The structural analysis of the first reported Nek2 inhibitor, SU11652, an ATP-competitive tyrosine kinase inhibitor with cross-reactivity toward several serine/threonine kinases, emerged as the lead guiding factor for Nek2 inhibitor development [140,183,184,185,186]. Crystal structure analysis of SU11652 complexed with inactive T175A-Nek2-mutant showed that it shares many common binding features of the typical kinase ligand complex. SU11652 occupies the back of the ATP binding cleft and stabilizes the T175A-Nek2-mutant kinase domain via a network of hydrogen bonding and van der Waals interactions (Figure 7). The pyrrole-indoline ligand engages in hydrogen bond interactions with the main chain atoms of Cys89 and Glu87 that lie within the hinge region. Additionally, the hydrophobic interactions between the side chain of Val68, Met86, Cys89, Phe148, Leu162 residues, and SU11652 further confers conformational stability on the protein. Interestingly, the pyrrole ring of the ligand forms an essential intramolecular hydrogen bonding with the indole amide that helps to preserve the planar geometry of the ligand.

SU11652 showed modest Nek2 inhibition with an IC_50_ value of 8 µM [Table 1] when determined using the peptide substrate GTFRSSIRRLSTRRRY (*K_m_* ~ 90 µM, *k_cat_* ~ 17 min^−1^). Interestingly, SU11248—a structural analog of SU11652—exhibited a marginally weaker inhibition profile [Table 1]. This is attributed to the smaller van der Waals radius and increased polarity of the fluorine atom [140]. Although SU11652 did not emerge as a potential therapeutic candidate, it certainly provided a wealth of information about targeting the Nek2 kinase that could potentially be utilized to develop better inhibitory scaffolds. The authors thus postulated that the incorporation of bulky residues in place of the chlorine atom in SU11652 will enable the exploitation of prospective allosteric interactions in the vicinity of ATP binding site. In addition, when considering the favorable interaction between the chlorine atom of the SU11652 and Leu162 of the protein, the authors considered the possibility of harnessing favorable interactions by targeting the surface of the Nek2 α-T that could potentially pave the way for finding a better therapeutic candidate.

### 8.2. Thiophene-Based Ligands

The thiophene-based motif garnered recognition as a potential Nek2 inhibitory scaffold during a drug discovery effort aiming to target the polo-like kinase 1 (Plk1) [141]. The Plk1 kinase, like Nek2, is a serine/threonine kinase involved in cell cycle regulation [187]. A library of thiophene-based inhibitors (Entry 2, Table 1) of Plk1(IC_50_: 2 nM), when evaluated against a panel of kinases, exhibited promising Nek2 inhibition. Although none of these compounds showed selectivity towards the Nek2 kinase, the findings turned out to be crucial for the development of Nek2-selective inhibitors. This will be further elaborated in the later sections.

### 8.3. Viridin/Wortmannin-like Ligands

High-throughput screening played a major role in Nek2 inhibitor discovery as Hayward et al. screened approximately 73,000 compounds and identified viridin/wortmannin-like compounds—known ATP site binding inhibitors of other kinase family members—as potential Nek2 inhibitors [142,188,189]. Two of this class of compounds (Entry 3a–b, Table 1) potently inactivated the Nek2 kinase and showed 70- to 1000-fold selectivity over Nek6 and Nek7 kinases, respectively. However, neither of these compounds showed any comparable selectivity when evaluated against Aurora A, Plk1, and Cdk1 mitotic kinases [142]. These compounds were cell-active, as both compounds **3a** and **3b** exhibited antiproliferative activity toward osteosarcoma U2OS cells and cervical cancer HeLa cells. Viridin analogs also negatively regulated premature centrosome splitting in Nek2A-inducible U20S cells. The authors further treated the same cell line with the selective Cdk2 inhibitor, as Cdk2 is known for inducing premature centrosome splitting [190]. Interestingly, they did not observe the same phenotype which indicated that the inhibition of cellular Nek2, and not Cdk2, by viridin analogs restricted the premature centrosome separation in the human tumor cell line. Though the authors did not evaluate the mode of inhibition of the Nek2 kinase by these analogs, they postulated that viridin analogs could potentially trap appropriately positioned lysine residue in the binding pocket. Recent studies have predicted K37 as a prospective residue for targeting and designing covalent inhibitors as seen in case of the PI3 kinase [191,192,193]. The authors further justified their hypothesis as they observed relatively higher Hill slope numbers (~1.5) for viridin analogs when compared to known reversible and ATP site binding inhibitor staurosporine (~1). Even though viridin analogs suffer from a promiscuous inhibitory profile, the study presented valuable insights on pharmacological inhibition of the Nek2 kinase and established it as a potential pharmacological target for cancer drug discovery.

### 8.4. Aminopyrazine Inhibitors

**Figure 8:**
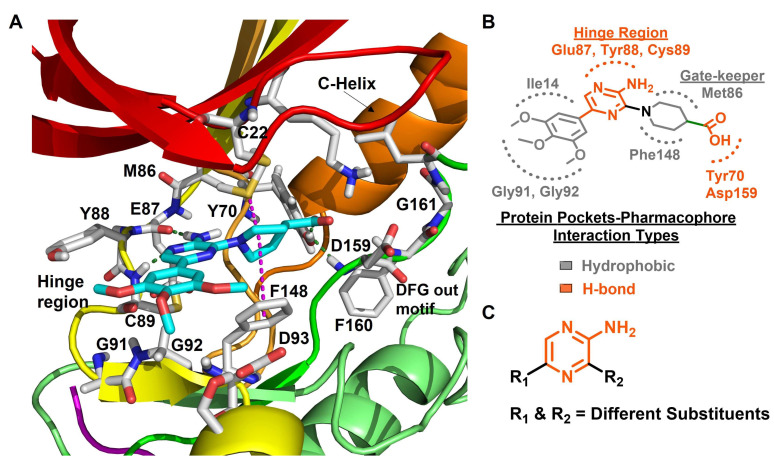
(**A**) Crystal structure of AP-1 and Nek2 complex. Trimethoxyphenyl group occupies the space between Ile and glycine-rich loop (G91 and G92). The piperidine ring is sandwiched between Phe148 and Met86, whereas the carboxyl establishes a H-bonding network between Tyr70 and Asp159. Aminopyrazine also establishes H-bonding interactions with the hinge region. All polar interactions are depicted by green dotted lines, whereas non-polar interactions are presented as purple dashed lines. (PDB code: 2xkf). (**B**) Schematic representation of AP-1 binding to Nek2, in which protein-ligand interactions have been designated using different color schemes. (**C**) Generic structure of 2-aminopyrazine analogs.

In a noteworthy study, Whelligan et al. discovered a new class of chemical phenotype, which—for the first time—inhibited both phosphorylated and unphosphorylated forms of the Nek2 kinase [143]. The authors initially performed a high-throughput screening and identified an aminopyrazine scaffold as a potential Nek2 inhibitory motif which they further adopted for thorough SAR studies. The initial lead compound AP-1 (Figure 8) exhibited a modest inhibitory profile toward the Nek2 kinase (IC_50_: 0.87 ± 0.34 µM) but suffered from low cell-permeability. This was attributed to the presence of readily ionizable free carboxyl group along with relatively high topological polar surface area. The authors primarily altered the substitutions at 2- and 6-positions of the aminopyrazine ring to address the issues associated with the lead compound. Crystal structure analysis of the unphosphorylated Nek2-AP-1 complex showed that it inherits many attributes of common kinase-inhibitor interactions (Figure 8). However, the ligand induced an unforeseen subtle change in protein structure, referred to as “Tyr-down” conformation. This phenomenon was also observed for few other analogs of this series of molecules. Interestingly, this class of compounds was found to inhibit both phosphorylated, i.e., active, and unphosphorylated, i.e., inactive, forms of the Nek2 kinase without much of a bias. The most potent analog of this series (Compound **4**, Table 1) exhibited a respectable inhibitory efficacy (IC_50_: 0.23 µM) and showed remarkable selectivity over Chk1—a kinase involved in cell-cycle regulation that does not share structural similarity with the Nek2 kinase. In addition, compound **4** exhibited > 80-fold selectivity over Plk1 (19.4 µM). However, it displayed compromised selectivity when evaluated against either form of the phosphorylated or unphosphorylated Nek1 (IC_50_: 0.17 µM) kinase. Finally, most of the developed aminopyrazine derivatives failed to exhibit improved cell-permeability. Overall, the study revealed a potential inhibitory scaffold for targeting the Nek2 kinase and provided valuable information on ligand-induced protein conformation that has been leveraged for future inhibitor discovery and will be discussed in upcoming sections.

### 8.5. Benzimidazole Inhibitors (DFG-Out)

**Figure 9:**
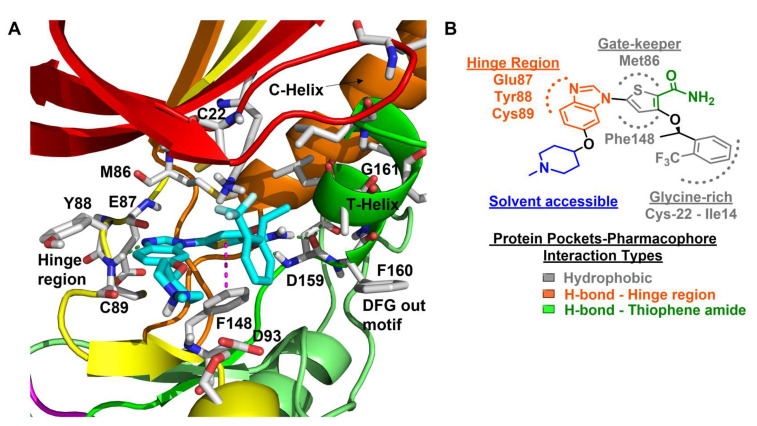
(**A**) Crystal structure of rac-1 bound inactive Nek2 that adopts a DFG-out conformation. Key protein residues and region that interact with the inhibitor have been designated. All polar interactions are depicted by green dotted lines, whereas non-polar interactions are presented as purple dashed lines. (PDB code: 2XNM) (**B**) Schematic representation of rac-1-bound Nek2 with color-coded interactions.

Benzimidazole has long been recognized as a preferred motif for kinase-related inhibitor discovery, which is known to engage in H-bonding interactions with the hinge region of the kinases [194]. In line with the observation, rac-1—a Benzimidazole-type compound—emerged as a favorable Nek2 kinase inhibitory scaffold (Figure 9) during an inhibitor discovery effort targeting the Plk1 kinase [141]. Solanki et al. thus adopted benzimidazole as a core motif and performed a thorough SAR analysis to construct a library of compounds that were subsequently evaluated to discover the pharmacophores with improved potency for the Nek2 kinase [144]. Their study revealed that substitutions at both thiophene and benzimidazole are prerequisites for effectively targeting the Nek2 kinase, as removal of either of them led to compounds with weaker inhibitory profiles. They also noted that the presence of a basic group, N-methyl piperidine, for instance—has a profound impact on dictating Nek2 inhibitory activity, as it positively cooperates with substituted phenyl groups (Figure 9). The authors further solved a rac-1 bound unphosphorylated Nek2 crystal structure that revealed many common interactions, which are hallmarks of typical kinase ligand interactions. In addition, the structure also captured the ligand-induced DFG-out conformation of the Nek2 kinase for the first time. The structure revealed that unsubstituted nitrogen of benzimidazole latched onto the hinge region by forming a hydrogen bond with the Cys89 residue. The thiophene moiety was sandwiched between Phe148 and the gatekeeper residue, Met86. Although the structure adopted a DFG-out conformation, the ligand possessed a unique binding mode, not commonly observed for other known DFG-out inhibitors. Nek2, unlike many other kinases, possesses a larger gatekeeper residue, Met86 that obstructed rac-1 from extending itself into the back pocket. The carbonyl oxygen of ligand established hydrogen bonding interactions with only the Asp159 of the DFG segment of the Nek2 kinase, unlike other ligands, which form an additional hydrogen bond with the catalytic glutamate residue of other kinases. The substituted benzyl group occupied the hydrophobic pocket formed by Cys22 and part of the glycine-rich loop (Ile14 and Gly15). Interestingly, only the *(R)* isomer of the racemic inhibitor, rac-1, was observed in the crystal structure which indicated that (*R*) configuration is favored over the (*S*) counterpart for targeting the Nek2 kinase. Taking clues from the above observation, the authors further performed computational studies and developed a series of compounds by retaining the benzimidazole core but replacing the thiophene with a phenyl ring. Although the most potent analog of this series, compound **5** (Entry 5, Table 1) failed to supersede the inhibitory efficacy of the parent analog, rac-1; it showed a remarkable 143-fold selectivity over Plk1 kinase. The co-crystal structure of the compound **5** bound to the Nek2 kinase revealed that the newly developed benzimidazole analog also induced a DFG-out conformation and engaged in similar interactions with the protein with few minor alterations; e.g., the piperidine ring occupied the back end of the catalytic groove and stayed on top of Gly91-Gly92 residues of the hinge region. Moreover, both rac-1 and compound **5** inhibited both the unphosphorylated and phosphorylated forms of the Nek2 kinase with comparable potency. Although compound **5** showed a modest selectivity profile against 24 different kinases, it showed cross-reactivity toward a few other kinases. Despite its good cell-permeability and modest metabolic stability, compound **5** failed to show significant cellular activity; this was attributed to elevated cellular ATP levels. It also manifested toxicity at higher concentrations owing to its non-selective inhibitory profile. Although this series of compounds failed to yield a therapeutic candidate, it exemplified the inhibition of Nek2 by inducing DFG-out conformation for the first time. This discovery had a significant impact on Nek2 inhibitor development, which was duly utilized in later studies.

### 8.6. Oxyindole-Propynamide Inhibitors

**Figure 10:**
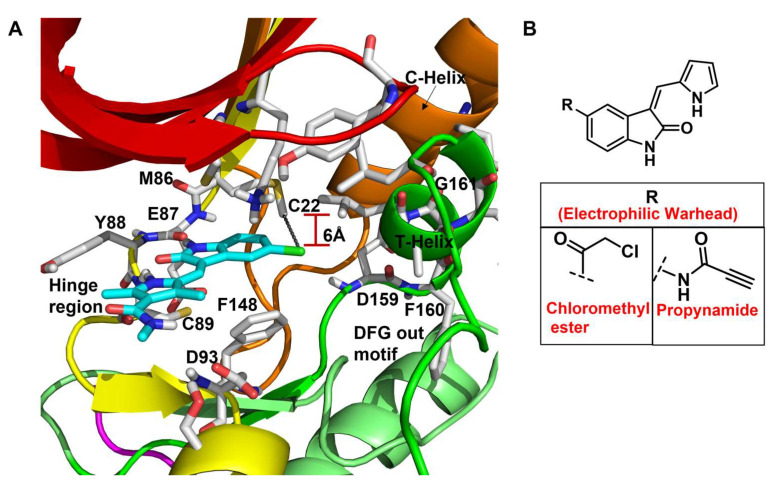
(**A**) The crystal structure of SU11652 bound Nek2 (PDB code 2JAV) revealed a distance of ~6 Å between Cys22 of the protein and the chlorine atom of the ligand, which had been utilized to design and construct new set of irreversible inhibitors. (**B**) Structures of the initial set of developed inhibitors that utilize electrophilic warheads for inactivating the protein.

In a seminal study, Henise et al. developed propynamide-containing irreversible inhibitors of the Nek2 kinase [145]. The targeting of non-conserved and non-catalytic cysteine residue not only emerged as a successful strategy for developing selective kinase inhibitors and useful chemical tools but also led to the development of multiple therapeutic candidates that successfully reached clinical trials for various indications [195,196,197,198,199,200,201,202,203]. A kinome-wide bioinformatics analysis showed that Nek2 and only 10 other kinases possess a targetable, non-conserved cysteine residue suitable for covalent inhibitor development. A testament to this hypothesis was the development of a selective irreversible inhibitor of Rsk1/2/4 [199,200,204]. The authors utilized this concept and developed a series of compounds by modifying a previously known oxyindole-based Nek2 kinase inhibitor and incorporating various electrophilic warheads for effectively hijacking the Cys22 residue of the Nek2 kinase. Interestingly, chloromethyl ketone and propynamide—both electrophilic warheads—exhibited maximum inhibitory efficacy compared to other electrophilic warheads (Figure 10). Both inhibitors successfully alkylated the wild-type Nek2 kinase as detected by electrospray mass spectrometry assay but failed to inhibit or alkylate the C22V Nek2 mutant. This demonstrated that this class of compounds inactivated the Nek2 kinase by effectively forming a covalent bond with the targetable non-conserved Cys22 residue. To aid the selectivity profile, authors further built two sets of compounds, retaining either chloromethyl ketone or propynamide as an electrophilic warhead and galvanizing differently substituted imidazoles. Among this new series of compounds, compound **6** inhibited Nek2 activity with a sub-micromolar IC_50_ value and showed improved selectivity over Rsk1, Plk1, and Cdk1 kinases. In addition, compound **6** also inhibited the Nek2 (IC_50_: ~1.3 µM) kinase in A549 human lung cancer cell lines but lacked efficacy against the C22V Nek2 mutant. Interestingly, compound **6** did not influence mitosis events, which further showed that the inhibitor did not interfere with other regulatory proteins—such as Cdk1, Plk1, AurB, and MPS1. Overall, this study presented a small molecule irreversible Nek2 inhibitor for the first time that not only shed light on the cellular roles of the Nek2 function but also provided the premise for developing Nek2-selective inhibitors, targeting the non-conserved Cys22 residue.

### 8.7. Aminopyridine Inhibitors

**Figure 11:**
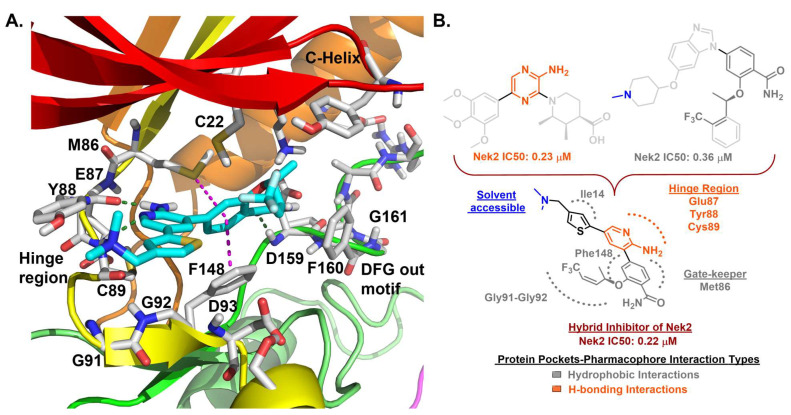
(**A**) Crystal structure of compound **7** bound Nek2 which shows the DFG-out conformation of the protein. Key H-bond and hydrophobic interactions are shown as green and purple dotted lines, respectively (PDB code: 4AFE). (**B**) The amalgamation of 2-aminopyrazine- and benzimidazole-type ligands leads to the construction of a new inhibitory motif for targeting Nek2 with improved efficacy.

Innocenti et al. duly realized the certain shortcomings of the existing 2-aminopyrazine- and benzimidazole-based Nek2 inhibitors and, thus, constructed a novel series of hybrid chemotypes that preserved and stimulated certain key structural features of the inhibitor classes discussed above (Figure 11) [143,144,146]. By performing a thorough SAR analysis, they designed compound **7**, which was comprised of: (a) 2-aminopyridine—a moiety known to interact with the hinge region by establishing two hydrogen bonds with Glu87 and Cys89 residues; (b) substituted benzamide, in which the phenyl ring formed a sandwiched interaction between the gatekeeper Met86 and Phe148 residues and the amide group forming hydrogen bonding interactions with the Asp159 of the DFG-motif; (c) allylic ether, which occupied a hydrophobic pocket formed by part of the glycine-rich loop; and (d) substituted thiophene that engaged in additional hydrophobic interactions with the Gly92 (Figure 11) and Ile14 residues (shown in schematic representation Figure 11B but not visible in Figure 11A, as it is buried under the beta-sheets). From the developed compounds, the (*R*) isomer inhibited Nek2 kinase activity with a low nanomolar IC_50_ value (Table 1, Entry 7, compound **7**) and exhibited remarkable selectivity over other mitosis regulatory kinases, such as Plk1, MPS1, AurA, and CDK2. In addition, 7 maintained its selectivity profile over a panel of 24 other kinases, except for a few non-cell cycle related ones. When tested in osteosarcoma U2OS cells, compound **7** inhibited C-NAP 1 phosphorylation, a physiological substrate of the Nek2 that is phosphorylated at S2179 position, in a dose-dependent manner and exhibited modest GI_50_ values against different cell lines. Although this inhibitor exhibited high permeability at physiological pH, as confirmed by parallel artificial membrane permeability assay, it suffered from high metabolic degradation. The developed Nek2 pharmacophore exhibited several positive traits of a good inhibitor, such as high potency and selectivity with improved cell-permeability, and it certainly added substantive values to the existing armory of Nek2-focused drug discovery efforts.

### 8.8. Quinoline-Based Inhibitors

Our laboratory took a holistic approach to discover and assess the efficacy of drug-like compounds for targeting Nek2 kinase activity. This approach involved first utilizing a rational bio-informatics-based approach to discover drug-like Nek2 inhibitory lead compounds, followed by assessing the efficacies and toxicities of newly identified pharmacophore using a whole animal-based Nek2 overexpression model [69]. Our hypothesis was that by following such a strategy, we would discover a non-toxic inhibitory pharmacophore that could be suitable for future drug development endeavors using animal-based pharmacology. This would also allow us to investigate perturbed Nek2-mediated signaling *in vivo* at the molecular level and help SAR optimization for enhanced anti-cancer activities. As described earlier, our developed whole animal Nek2 overexpression model in flies led to the deregulation of several cell migration markers—Rho1, Rac1, and Ecad—along with unique tissue phenotypes, amenable for drug screening *in vivo*. dNek2 overexpression, in conjunction with receptor tyrosine kinase and mitogen-activated protein kinase, promoted local invasion, distant cell seeding, and metastasis. The Nek2 kinase was established as a key mediator that cooperated with other oncogenic signaling pathways to promote aggressive colonization of tumorigenic cells into adult fly tissues. With the goal of identifying a novel type of non-toxic Nek2 pharmacophore, we undertook a bioinformatics-based computational approach. We analyzed the primary sequence and crystallographic structures of the Nek2 kinase and noted the presence of a unique helical motif (αT), just outside of the DFG motif, which was also present in receptor tyrosine kinases, EGFR and HER2 [69]. Although none of the three kinases exhibited overall significant sequence identity or similarity, comparison of tertiary structures of ATP site-directed inhibitor-bound complexes revealed that they shared analogous tertiary folds involving common key interactions. This led us to hypothesize that certain EGFR/HER2 inhibitory candidates could also inhibit Nek2 activity potently. We then implemented a computational screening protocol *in silico* on an existing library of advanced-stage clinical inhibitory agents of EGFR/HER2 kinases and discovered that pelitinib (EKB-569) and neratinib (HKI-272) (Table 1, Entry 8) inhibited Nek2 kinase activity potently. Interestingly, despite the presence of a Michael acceptor (crotonamide moiety), both compounds inhibited Nek2 reversibly, as evaluated using *in vitro* recombinant enzyme assays. In addition, both pelitinib and neratinib effectively suppressed the Nek2-driven distant seeding phenotype in a dose-dependent manner in flies. Further validation of cellular Nek2 inhibition by this class of molecules was established in human lung adenocarcinoma A549 and HEK293T cells. Quite interestingly, pelitinib with a weaker *in vitro* inhibitory profile than its peer neratinib emerged as a better pharmacophore in suppressing Nek2-mediated signaling in the *in vivo* fly model, further underscoring the importance of the inclusion of *in vivo* models in early drug discovery endeavors. This study articulated the role of the Nek2 kinase in promoting metastasis and showed the significance of its pharmacological inhibition in development of anticancer therapeutics. Our investigation also revealed for the first time that targeting multiple effectors in conjunction with the Nek2 kinase may present an attractive alternative for treating diseases where the involvement of this kinase has been ascertained. 

### 8.9. Pyrimidine Inhibitors

An investigation by Zhou et al. indicated that Nek2 kinase overabundance resulted in poor prognosis, rapid cell proliferation, and drug resistance in multiple myeloma [68]. Almost one-third of the patients with relapsed multiple myeloma (MM) either did not respond or developed resistance towards bortezomib, the primary treatment option for relapsed refractory multiple myeloma. Following this lead, Meng et al. studied the effect of Nek2 inhibition, in conjunction with proteasome inhibition, on the progression of multiple myeloma [147]. The authors screened about 2000 chemical candidates and discovered hit compounds, among which HCI-2389 (Table 1, Entry 9) exhibited maximum inhibitor potency towards the Nek2 kinase. The authors demonstrated that ectopic expression of Nek2 stimulated bortezomib resistance in HeLa cells and boosted proteasome activity in bortezomib-resistant ARP-1 cells. This was, however, reversed upon HCI-2389 treatment. Furthermore, HCI-2389 in combination with bortezomib stalled proteasome activity much more effectively compared to either drug when administered individually. At the molecular level, overexpression of Nek2 led to the downregulation of Cyclin B and Cdc2 via proteasome-mediated degradation which was reversed upon inhibitor treatment. On the mechanistic front, the inhibitor HCI-2389 contained a Michael acceptor type acrylamide moiety and possibly modified the protein covalently, as found by the time-dependent enhancement in inhibitory efficacy. In summary, this study presented a viable strategy of inhibiting the Nek2 kinase for a better outcome in bortezomib-resistant MM patients. A subsequent investigation by Franqui-Machin et al. also showed that Nek2 kinase destabilization overcame resistance to proteasome inhibition in MM [205]. 

### 8.10. Imidazo[1,2-a] Pyridine Inhibitors

While significant progress was made towards developing Nek2 inhibitors thus far, a lack of desirable *in vivo* results barred any of the developed scaffolds from being considered viable clinical candidates. Fang et al. addressed this issue by discovering a new compound, MBM-5 [Table 1, Entry 10], which exhibited promising *in vivo* activity [148]. The authors initially screened 500 compounds and identified MBM-5 as a lead compound, with sub-micromolar IC_50_ value towards the Nek2 kinase. The computational study predicted that MBM-5 would occupy the ATP-binding site and establish a hydrogen bonding interaction with Cys89 and Asp159 residues, along with additional pi-stacking interaction with Phe148 residue. The cell-based assay revealed that MBM-5 effectively reduced Hec1 phosphorylation and, even more intriguingly, led to reduced levels of Hec1 protein. Notably, MBM-5 did not affect the Aurora A kinase activity. When tested on multiple cancer cell lines, MBM-5 was found to be more effective towards leukemia, gastric, and colorectal cancer cell lines as the EC_50_ values were in the low micromolar range. In line with other Nek2 inhibitors, MBM-5 caused mitotic abnormalities and induced apoptosis. Most importantly, MBM-5 exerted significant efficacy in suppressing cancer growth in MGC-803 gastric and HCT-116 xenografts mice. Although MBM-5 showed some promising traits of a desirable clinical candidate, it suffered from low half-life *in vivo,* barring its advancement to the next level. Despite this, MBM-5 represented a new scaffold for Nek2 selective inhibitor design that could potentially be exploited for developing an improved pharmacophore in the future.

In an interesting study, Wang et al. noted that Nek2 acts as a functional protein binding partner of enhancer of zeste homolog 2 (EZH2) and post-translationally regulates EZH2 in glioma stem cells (GSCs) [149]. Nek2 phosphorylates EZH2 upon binding and protects it from ubiquitination-dependent protein degradation. This, in turn, manifests tumor propagation and radioresistance, leading to poor prognosis in GSCs. To investigate the clinical relevance of Nek2 inhibition, the authors resorted to developing Nek2 selective inhibitory chemotypes. They initially screened 300 chemical candidates and found a lead compound that, upon suitable optimization, as assisted by the computer-based drug discovery, yielded compound CMP3a (Table 1, Entry 11). This compound potently inactivated the Nek2 kinase and showed remarkable selectivity over a panel of about 100 kinases. CMP3a showed remarkable efficacy in sensitizing GSCs, cells with elevated Nek2 expression levels, compared to normal human astrocytes. Importantly, intravenous administration of CMP3a effectively silenced tumor growth in GSC-derived xenotransplanted mice with no observable off-target effects. CMP3a is the first chemotype of its kind to induce proteasome-mediated EZH2 degradation. However, unlike many other small molecule Nek2 inhibitors, CMP3a did not cause chromosomal instability in GSCs. Noticeably, CMP3a treatment showed a synergistic effect with radiation and effectively decreased radio-resistance of the glioma spheres in a dose-dependent manner and not in normal human astrocytes (NHAs). Although CMP3a represented a unique mode of action for sensitizing GSCs with elevated Nek2 levels and presented a promising option for future cancer therapies, it did not have a favorable pharmacokinetic profile (a short half-life), and thus failed to advance in clinical trials.

### 8.11. Purine Inhibitors

**Figure 12:**
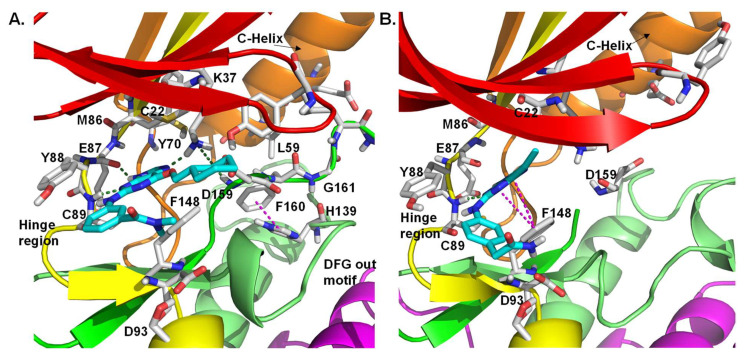
(**A**) Crystal structure of the purine inhibitor, compound **12a** bound to T175A Nek2 kinase. The R-spine forming residues—Tyr70, Leu59, Phe160, and His139—are shown in magenta. Pi stacking interaction between Phe160 and His139 is shown by the dotted line (magenta). Inhibitor engages in H-bonding interaction (dotted green) with the hinge region (orange ribbon) in addition to Lys39 (blue) which further interacts with Asp159 (blue) of DFG. (PDB code: 5M53) (**B**) Crystal structure of compound **13** bound to Nek2 kinase. Cys22 makes a covalent bond with the alkyne group, whereas the purine ring occupies the ATP-binding site and forms H-bonding with the hinge region (shown as a green dotted line) in addition to the pi-stacking interaction that it experiences from Phe148 (shown as a magenta dotted line). (PDB code: 6SGH).

Purine-based pharmacophores were introduced by Coxon et al. as ATP-competitive reversible inhibitors of Nek2 and CDK2 kinases [150]. After performing a thorough SAR study, the authors developed a series of Nek2 inhibitors with significant selectivity over the CDK2 kinase. This was accomplished by judiciously optimizing the purine core. Crystal structure analysis of one of the purine analogs—compound **12a** (Figure 12A) bound to the inactive T175A Nek2 mutant—represented the first example of Nek2 inhibition with DFG-in and α-C helix out conformation. This ligand interacted with Lys37 residue, establishing a connection with the DFG motif via a hydrogen bonding interaction with the side chain of Asp159. In addition, it enforced a more ordered R-spine in the Nek2 kinase, which was reminiscent of the active state of the protein in which the Phe160 of the DFG motif engages in a pi-stacking interaction with the histidine of the HRD motif (Figure 12A). Further modification of this scaffold led to the discovery of the most potent analog of this series (Table 1, Compound **12b**), which exhibited 10-fold selectivity over the CDK2 kinase. This compound was also found to be effective in growth inhibition assays involving U2OS, MDA-MB-231, and HeLa cell lines. Unfortunately, 12 was found to be unstable under simulated physiological conditions; thus, it could not progress further as a clinical candidate.

A study by Lebraud et al. had shown that the Michael acceptor containing ethynylpurine analogs can effectively form a covalent bond with the nucleophilic thiol of N-acetyl cysteine methyl ester and, thus, could potentially trap non-conserved Cys22 of the Nek2 kinase; this could provide an opportunity to develop more potent covalent inhibitors with higher residence time [206]. Matheson et al. aptly adopted this strategy and developed a series of 6-ethynylpurine derivatives as irreversible inhibitors for targeting Nek2 [151]. The authors utilized a model inhibitory compound **13** (Table 1, Entry 13) for subsequent biological evaluation. This compound exhibited promising selectivity over a panel of other kinases and showed moderate efficacy in a growth inhibition assay in several human solid tumor and leukemia cell lines. Compound **13** also inhibited C-Nap1 phosphorylation, a Nek2 substrate, in U2OS cell lines in a dose-dependent manner. The authors further evaluated the mode of inactivation and the binding pattern of the ligand to the Nek2 kinase. The compound was devoid of activity when treated with the A22C Nek2 mutant, demonstrating the necessity of nucleophilic non-conserved cysteine residue for Michael addition. Crystal structure analysis of the ligand-bound Nek2 complex further corroborated the previous observation that the triple bond of purine was the site of action of the non-conserved Cys22 residue (Figure 12B). Compound **13** shared some common interactions with its reversible counterpart; it occupied the ATP-binding site and engaged in a H-bonding interaction with the hinge region. It did not however impose the R-spine organization in the Nek2 (see Figure 12 A) kinase. Finally, the newly developed compound failed to exhibit a desirable pharmacokinetic (PK) profile either *in vitro* or *in vivo*. Although the newly developed core falls short of becoming a therapeutic candidate, it provides ample knowledge that could be leveraged for future drug discovery efforts targeting the Nek2 kinase.

### 8.12. Protein-Protein Interaction (PPI) Inhibitors

Several key studies have revealed the important features of Nek2-associated protein-protein interactions as key regulatory events during mitosis [12,36,42,44]. PPI proved to be a significant finding from the point of view of Nek2 drug discovery and was leveraged for the first time by Wu et al [45]. The authors, in their pursuit of finding Hec1 selective inhibitors, screened ~24,000 compounds and discovered two 2-aminothiazole derivatives that interrupted Hec1/Nek2 binding. Subsequent studies with one of the lead inhibitors (INH1, Table Entry 14) revealed that the thiazole derivative disrupted Hec1/Nek2 interaction by preferentially binding to the Hec1 protein. Interestingly, the treatment of cells with this class of compound led to a depletion of cellular Nek2 levels in a dose- and time-dependent manner. Compound **14** was also found to (a) inhibit tumor cell proliferation, (b) promote mitotic abnormalities, and (c) retard tumor growth. These findings are the hallmark of the inhibition of the Hec1/Nek2 axis. This study ushered an alternative approach to the targeting of Nek2 that was subsequently adopted for building improved Nek2 inhibitory phenotypes.

The identification of thiazole derivative as an Hec1/Nek2 disruptive PPI agent promoted further medicinal chemistry activities, leading to the identification of another functionalized thiazole, compound **15** (Table 1) that disrupted the Hec1/Nek2 interaction with improved efficacy as evaluated in several cell lines, including MDA-MB-231 and Hela [207]. This PPI inhibitor efficiently promoted chromosomal misalignment and caused cell death. This newly developed PPI inhibitor, compound **15**, provided the prospect of further refinement of this scaffold to yield therapeutic agents with an improved pharmacological profile.

**Figure 13:**
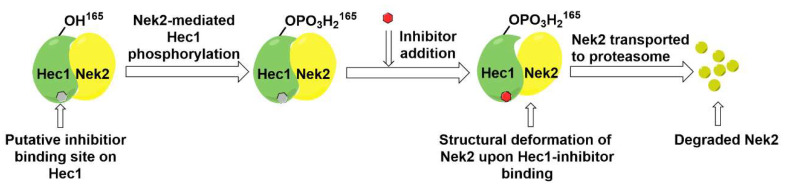
Schematic representation of targeting Hec1/Nek2 axis by thiazole-based inhibitors and its effect on promoting proteasomal degradation of Nek2 kinase.

In a follow-up study, Hu et al. developed the third generation of 2-aminothiazole derivatives that were more effective than their predecessors [47]. The most effective compound of this class, INH154, (Table 1, Entry 16) prompted mitotic abnormalities and subsequent cell death. In line with the previous observations, INH154 effectively reduced the Nek2 levels in human breast cancer cell lines, MDA-MB-468 and MDA-MDA-MB-231, and abolished Nek2-mediated HEC1-S165 phosphorylation. Interestingly, treatment of the cell lines with proteasome inhibitor before the thiazole derivative treatment rescued the protein level, consistent with the proteasome-mediated degradation of Nek2 at prometaphase [208]. Subsequent studies revealed that the interaction between the Ile408-Leu422 region of the Hec1 and R361 residue of the D-box (destruction-box) region of the Nek2 kinase is a prerequisite for 2-aminothiazole derivative-mediated Nek2 depletion (Figure 13). Considering the evidence, the authors proposed that Nek2 initially binds and phosphorylates Hec1 on S165 at M phase in cells; however, subsequent binding of thiazole moiety to Hec1 leads to conformational changes in the Nek2 kinase, ultimately ensuing proteasome-mediated Nek2 degradation. INH154 was further found to be effective in sensitizing cancer cell-lines with elevated levels of Hec1 and Nek2, as demonstrated by the suppression of tumor growth in mouse xenografts. The non-toxic nature of the compound further enhanced its pharmacological profile. Taken together, this study strongly posits that targeting both Hec1 and Nek2 could potentially be a viable strategy for the treatment of highly aggressive cancers.

While exploring the potential of the 2-aminothiazole scaffold as anti-tumor agents by derivatizing with other therapeutically relevant scaffolds, Lu et al. discovered a cinnamide-2-aminothiazole compound, TH-39, (Table 1, Entry 17) which exhibited anti-cancer activities in different cell lines with the EC_50_ values in low micromolar to nanomolar range [152]. Considering the structural similarity of the antiproliferative agents of this series with INH1, a Hec1/Nek2 inhibitor, Zhu et al. adapted TH-39 and set out to reveal the underlying mechanism associated with the compound [46]. TH-39 stalled cancer proliferation in K562 cells, a cell line with elevated Hec1/Nek2 levels. Importantly, TH-39 remained docile towards non-cancerous cells. Not surprisingly TH-39, like INH-analogs, affected Hec1/Nek2 interaction and promoted mitotic abnormalities. Further investigation revealed that TH-39 disrupted the mitochondrial membrane potential and invoked apoptosis by accumulating elevated levels of reactive oxygen species. Although TH-39 reinforced the viability of the 2-aminothiazole scaffold as a building block of the Hec1/Nek2 disruptor, the presence of the Michael Acceptor, acrylamide, poses a threat of invoking off-target effects. Thus, further mechanistic evaluation coupled with an *in vivo* toxicity study is required before promoting this compound to a clinical stage.

Huang et al. further explored the prospect of 2-aminothiazole compounds and discovered a novel analog, TAI-1 (Table 1, Entry 18), which exhibited a significantly improved pharmacological profile, compared to the earlier reported ones [153]. The newly developed compound preserved the previously observed mechanism of action towards Hec1 and Nek2 and sensitized a wide array of cancer cells including several multidrug-resistant cell lines, such as MES-SA/Dx5, NCI-ADR-RES, and K562R. TAI-1, when administered orally; it also effectively suppressed tumor growth in xenograft mouse models of triple-negative breast cancer, and colon and liver cancers, with no adverse toxic effects. Notably, the ligand also maintained a high therapeutic index as it (a) had little or no effect on normal cell lines, such as WI-38 or RPTEC, (b) did not show activity towards a panel of kinases, and (c) did not inflict cardiac toxicity by hERG. In addition, the pharmacophore also exhibited synergy with doxorubicin, topotecan, and paclitaxel in the breast, leukemia, and liver cancer cells. The efficacy of compound TAI-1 was, however, found to be impacted by the status of retinoblastoma and P53 levels. This study further propels the prospect of disrupting the nexus of Hec1 protein and Nek2 kinase as a viable strategy for future anticancer drug development.

The ongoing optimization of the thiazole core has, further, led to the discovery of a TAI-1 derivative, compound **19**, (Table 1) which contains an additional fluorine substitution at the ortho position of the pyridine ring (Table 1, Entry 19) [154]. This single substitution had a pronounced effect on the pharmacological profile of the developed pharmacophore, as it exhibited low nanomolar *in vitro* antiproliferative activity, high intravenous area under the curve (AUC) in Sprague Dawley (SD) rats and improved the tumor suppression ability in mice bearing human MDA-MB-231 xenografts. A relentless pursuit of developing a clinical candidate for destabilizing the Hec1/Nek2 axis has led to the discovery of TAI-95 (Table 1, Entry 20), a highly effective compound, when administered orally and/or intravenously, in suppressing tumor in mice bearing human MDA-MB-231, BT474, and MCF7 xenografts [155,156]. The compound TAI-95 also showed efficacy in treating liver cancer, as it significantly suppressed tumor growth in mice bearing human Huh-7 xenograft with oral administration. Considering the therapeutic potential of TAI-95, the compound was formulated to obtain T-1101 tosylate (Table 1, Entry 20), which not only exhibited improved oral pharmacokinetics and anti-cancer activity but also exhibited a robust synergistic anticancer effect when administered in conjunction with doxorubicin, paclitaxel, and topotecan [157]. This compound is currently being investigated in clinical trials as a potential anti-cancer therapy for sensitizing the HEC1/Nek2 axis.

## 9. Expert Opinion

The extensive research reported so far strongly suggests that the inhibition of Nek2 activity in diseased states may lead to successful therapeutics. The advent of several classes of small molecule Nek2 inhibitors have significantly broadened our prospect of developing novel Nek2-targeting therapeutic modalities. Despite the focused efforts over the last two decades, only one PPI inhibitor chemotype, T-1101 Tosylate, has made it to clinical trials. Given the involvement of the Nek2 kinase in several diseases, it would be important to further enrich the Nek2 inhibitory pipeline for successful clinical trials [157]. In this context, one should not only rely on the conventional strategies but should investigate other viable options that have proven beneficial for this class of enzymes. One such strategy is to develop selective covalent and reversible chemotypes targeting the non-conserved Cys22 of the Nek2 kinase. Such agents, like their irreversible counterparts, covalently modify the target proteins. However, they detach themselves from the target protein over time, thus maintaining the prolonged pharmacological effect, and yet shedding the potential immuno-liabilities associated with the permanent modification of the target protein [209]. Another conceivable strategy that has proven attractive for certain kinases, but not yet tested on the Nek2 kinase, is to pursue PROTAC-based modality. This strategy notably extends several advantages over classical small molecule inhibitory chemotypes. It (a) degrades the target protein along with its downstream signaling cascades over a longer period of time, (b) could overcome problems associated with on-target mutation-driven drug resistance, (c) enhances target selectivity and, (d) evades potential dose-limiting toxicities due to its sub-stoichiometric catalytic mode of action [210,211]. Other interesting strategies could include a polypharmacological approach, whereby one sensitizes multiple nodes of a biological signaling network, as shown by Dar and Das et al., to produce a more pronounced therapeutic efficacy than what can be achieved by inhibiting a single target by a single agent [212]. Concerns, however, remain over acquiring desirable therapeutic index in Nek2 kinase inhibition, primarily due to its ubiquitous presence and its involvement in a fundamental cell cycle process—mitosis (as a result, there are concerns about achieving inhibition selectivity between diseased and normal cells). It may thus be useful to undertake a pro-drug approach to target the Nek2 kinase only in diseased cells. This could be attained by adapting an optimal drug delivery carrier (e.g., an antibody-drug conjugate) for selective payload delivery, thereby minimizing off-target dose-limiting toxicities [213]. Another approach that could also aid the current drug discovery effort would be to develop selective activity-based Nek2 probes. Activity-based probes enable monitoring of protein activity in both *in vitro* and *in vivo* settings. They allow target identification and validation, proteome-wide analysis of off-targets, and subsequent analysis of signal transduction pathways [214]. This strategy may reveal unidentified modes of the Nek2 kinase interactome that could potentially be utilized to develop novel chemotypes. Given the Nek2 kinase’s involvement in several forms of aggressive cancers, and its role in promoting metastasis and drug resistance, it presents a unique opportunity for anti-cancer drug development. Availability of a robust direct continuous assay (e.g., a fluorescence- or UV-Vis-based assay) and a cell-active biosensor should further expedite the drug discovery process and drug validation efforts. Importantly, pairing these cell-based studies with whole animal model systems with sophisticated genetic toolkits such as flies, zebrafish, and mice will allow further mechanistic insights into the function of Nek2 and related pathways. Strategic use of these whole animal models will, as reviewed here, also provide a robust tool for a medium-throughput drug screening pipeline with key *in vivo* endpoints—the inhibition of Nek2 and related pathways with low whole animal toxicity [215,216,217]. We remain cautiously optimistic about the prospect of developing targeted therapeutics for the Nek2 kinase in the foreseeable future.

## Figures and Tables

**Table 1:** Small Molecule Inhibitors of Nek2 Activity and Their Cellular Profile.

Entry #	Chemical Structure	Inhibition Profile with the Compound I.D. (In Parenthesis)	Special Notes and Reference	Inhibitor Type
1	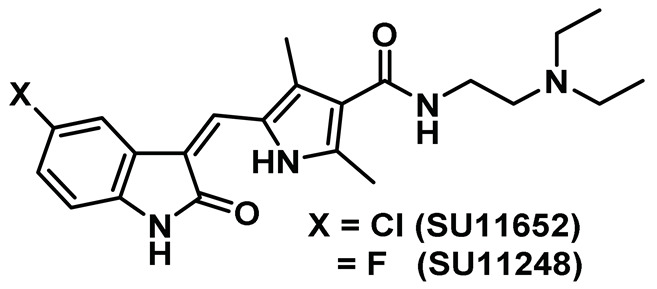	IC_50_ (SU11652): 8 µM IC_50_ (SU11248): 12 µM	The first reported non-selective and cell-permeable chemotype that binds to the inactive conformation of Nek2 [140].	Type II
2	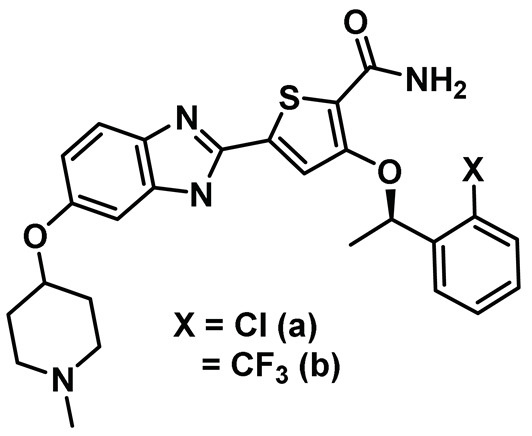	IC_50_ (2a): 21 µM IC_50_ (2b): 25 µM	Thiophene-based inhibitors, originally developed for targeting PLK1, emerged as potential Nek2 inhibitory scaffold [141].	NA
3	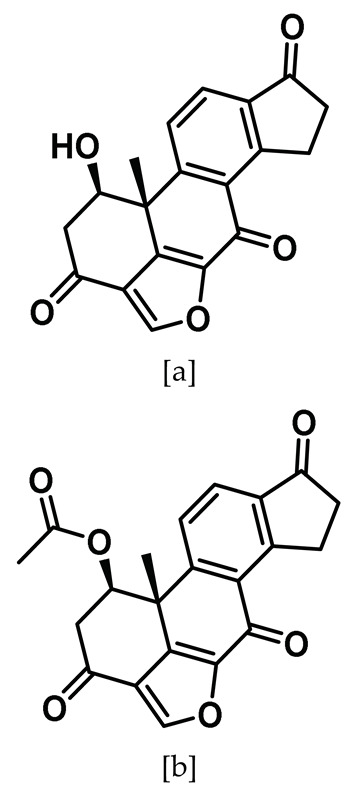	IC_50_ (3a): 1.4 µM IC_50_ (3b): 4.4 µM	Cell-active Nek2 inhibitors that showed significant Nek2 selectivity when compared to Nek6 and Nek7 but not toward Aurora A, Plk 1, and Cdk1. Affected centrosome separation in Nek2-inducible human tumor cells [142].	Type VI ^$^
4	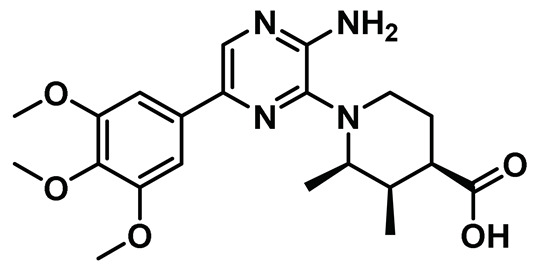	IC_50_ (4): 0.23 µM	Inhibits Nek2 by inducing notable conformational changes, “Tyr-down”, in protein. Non-selective and suffers from poor cell-permeability [143].	NA
5	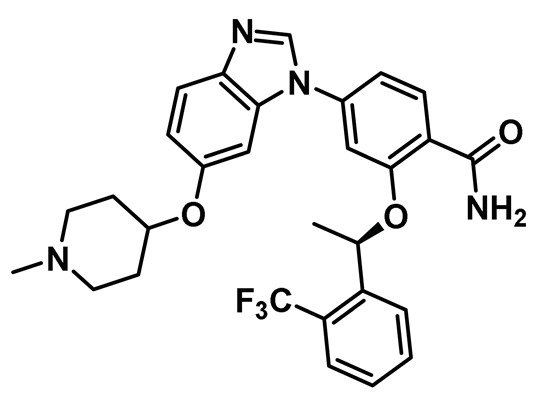	IC_50_ (5): 0.36 µM	The first benzimidazole-type inhibitor to induce DFG-out conformation in Nek2 kinase. Although it exhibited remarkable selectivity over Plk1, it failed to maintain the selectivity when tested against other kinases. It also suffered from the lack of cellular potency [144].	Type II
6	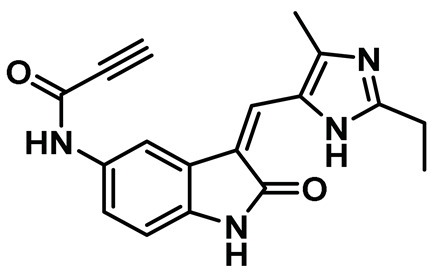	IC_50_ (6): 0.77 µM	The first developed irreversible inhibitor of Nek2 with cellular activity which covalently modifies the protein by trapping Cys22 [145].	Type VI
7	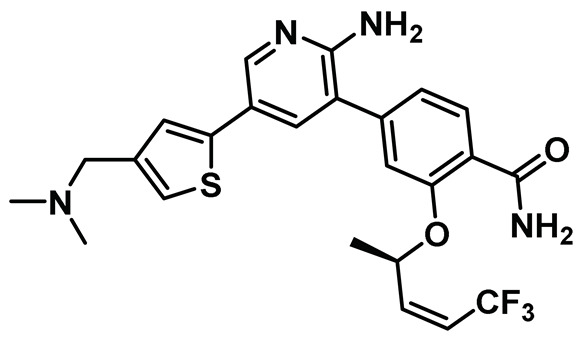	IC_50_ (7): 0.022 µM	A cell permeable Nek2 inhibitor with improved selectivity that induces a DFG-out conformation of protein [146].	Type II
8	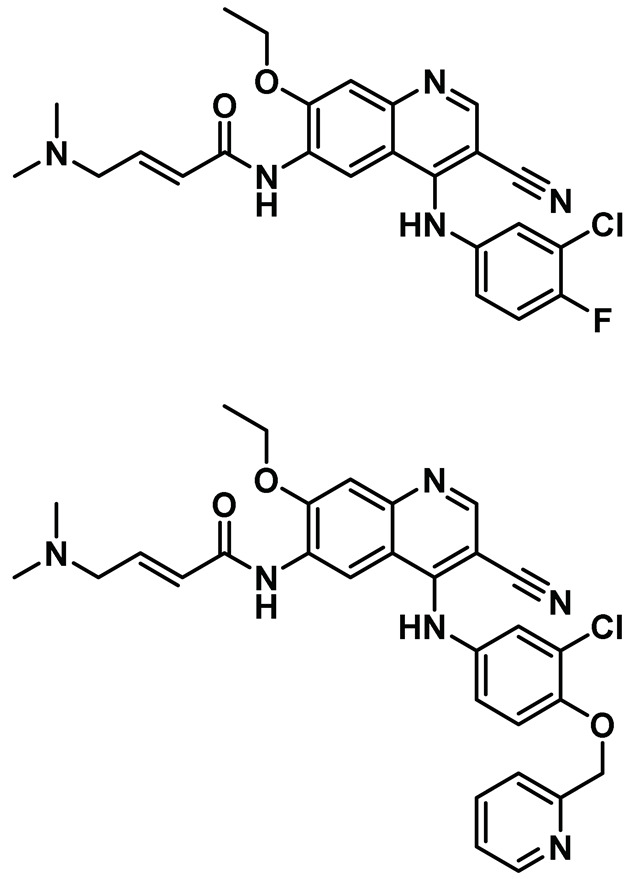	IC_50_: (Pelitinib/EKB-569): 661 nM IC_50_: (Neratinib/HKI-272): 247 nM	Using a whole-animal-based Nek2 overexpression model in flies, two cell active EGFR inhibitors were found to be active against Nek2 kinase [69].	NA
9	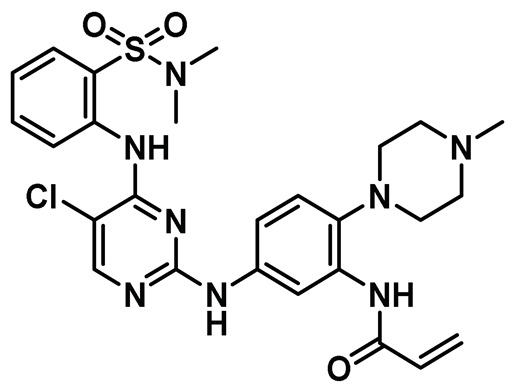	IC_50_ (HCI-2389): 0.016 µM	Highly potent and cell-active Nek2 inhibitor that effectively sensitized bortezomib-resistant multiple myeloma cells [147].	Type VI
10	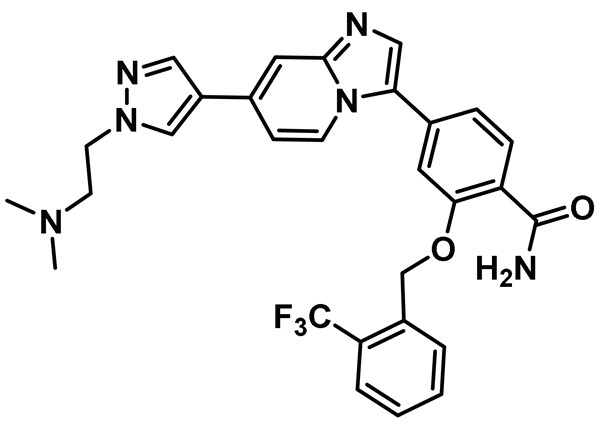	IC_50_ (MBM-5): 0.34 µM	Effectively inhibited Nek2 kinase in leukemia and gastric and colorectal cancer cell lines and retained its efficacy while being evaluated *in vivo* using MGC-803 gastric and HCT-116 xenografts in mouse models. Although promising, MBM-5 suffered from unimpressive pharmacokinetic profile [148].	NA
11	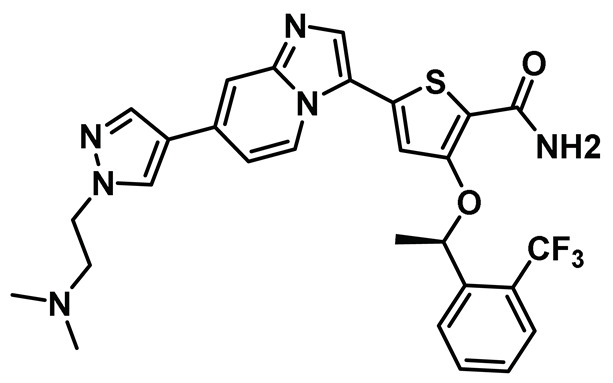	IC_50_ (CMP3a): 0.082 µM	Disrupts the Nek2-EZH2 nexus in glioma stem cells and silenced tumor in xenotransplanted mouse. Poor pharmacokinetic profile of CMP3a only allowed it to be used as a chemical tool and not as a therapeutic modality [149].	NA
12.	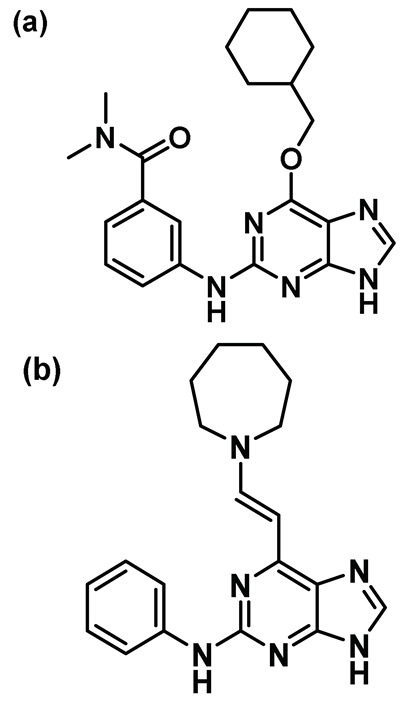	IC_50_ (12a): 0.62 µM IC_50_ (12b): 0.27 µM	First inhibitor of its kind to invoke DFG-in configuration in Nek2 kinase [150]. Most potent analog of this series. Despite notable potency, it failed to become a therapeutic candidate due to instability reasons under physiological conditions.	Type I 1/2
13	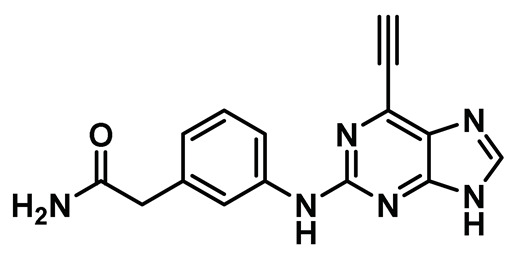	IC_50_ (13): 0.062 µM	A cell-active irreversible inhibitor of Nek2 kinase that showed modest selectivity profile over several other kinases; however, it lacked a desirable pharmacokinetic (PK) profile [151].	Type VI
14	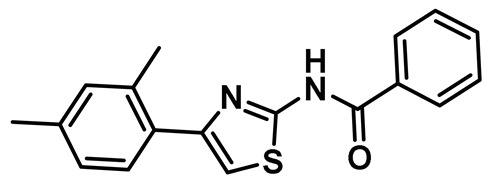	IC_50_ (INH1): NA	First discovered 2-aminothiazole-based PPI inhibitor of Hec1/Nek2 axis with cellular activity [45].	NA
15	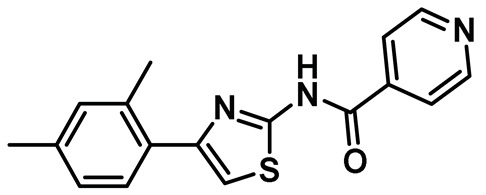	IC_50_ (15): NA	Second generation of cell-permeable thiazole derivatives that affected Hec1/Nek2 activity [47].	NA
16	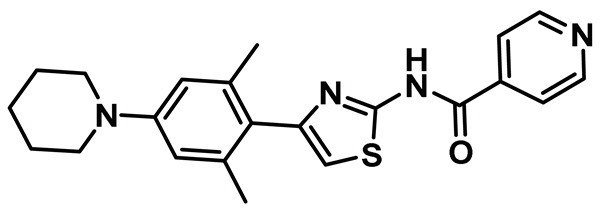	IC_50_ (INH154): NA	Third generation thiazole-derivative with little or no toxicity that suppressed tumor growth effectively in mouse xenograft model upon peritoneal administration [47].	NA
17	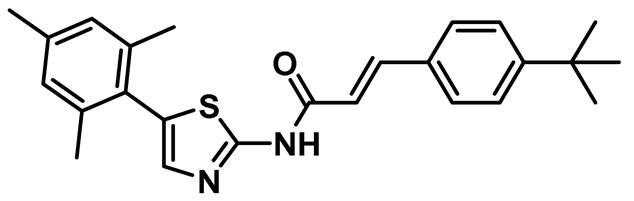	IC_50_ (TH-39): NA	A cell-active 2-aminothiazole derivative that exhibited the hallmark of Hec1/Nek2 inhibition [46,152].	NA
18	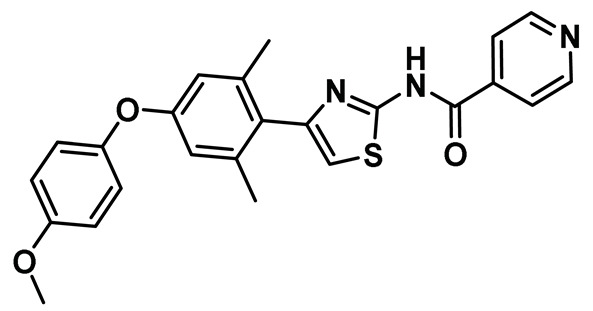	IC_50_ (TAI-1): NA	The first orally administered Hec1/Nek2 inhibitor—also effective when administered intravenously—with little or no adverse effect. It showed tremendous promise in suppressing tumor growth in mouse model [153].	NA
19	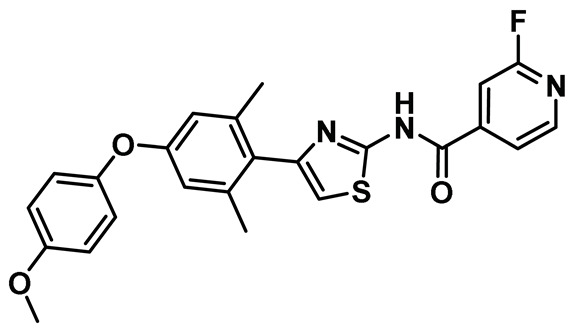	IC_50_ (19): NA	Extremely potent Hec1/Nek2 disruptor that presented a high AUC when administered in SD rats [154].	NA
20	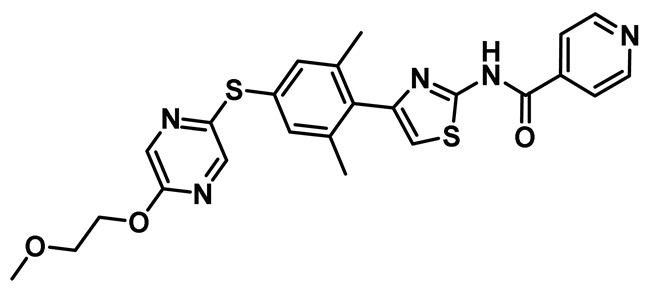 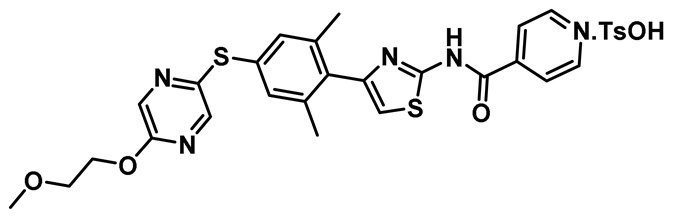	IC_50_ (TAI-95): NA IC_50_ (T-1101 tosylate): NA	A very potent HEC1/Nek2 inhibitor with impressive pharmacological profile that is currently being evaluated in clinical trials [155,156,157].	NA

^$^ Plausible inhibitor types. NA: Not available.

## Data Availability

Not applicable.
